# C-SMB 2.0: Integrating over 25 years of motor sequencing research with the Discrete Sequence Production task

**DOI:** 10.3758/s13423-023-02377-0

**Published:** 2023-10-17

**Authors:** Willem B. Verwey

**Affiliations:** Department of Learning, Data-Analytics and Technology, Section Cognition, Data and Education, Faculty of Behavioral, Management and Social sciences, University of Twente, PO Box 217, 7500 AE Enschede, the Netherlands

**Keywords:** Discrete sequence production task, Cognitive framework of sequential motor behavior, Motor learning, Movement sequences, Processing architecture

## Abstract

An exhaustive review is reported of over 25 years of research with the Discrete Sequence Production (DSP) task as reported in well over 100 articles. In line with the increasing call for theory development, this culminates into proposing the second version of the Cognitive framework of Sequential Motor Behavior (C-SMB 2.0), which brings together known models from cognitive psychology, cognitive neuroscience, and motor learning. This processing framework accounts for the many different behavioral results obtained with the DSP task and unveils important properties of the cognitive system. C-SMB 2.0 assumes that a versatile central processor (CP) develops multimodal, central-symbolic representations of short motor segments by repeatedly storing the elements of these segments in short-term memory (STM). Independently, the repeated processing by modality-specific perceptual and motor processors (PPs and MPs) and by the CP when executing sequences gradually associates successively used representations at each processing level. The high dependency of these representations on active context information allows for the rapid serial activation of the sequence elements as well as for the executive control of tasks as a whole. Speculations are eventually offered as to how the various cognitive processes could plausibly find their neural underpinnings within the intricate networks of the brain.

## Introduction

“*I called the talker, critic, controlling voice Self1 and the self that had to hit the ball Self2. It soon became apparent that the less controlling, judgmental conversation there was from Self 1, the better the shots would turn out.’’ (From The Inner Game of Tennis, *Gallwey, 1974/1997*)*

Intelligent, creative, and goal-directed behavior would be impossible if we would continuously be engaged in controlling every minute aspect of our behavior. Yet people appear to develop behavioral ‘building blocks’ that are stored in long-term memory (LTM) and that can be retrieved as a whole when performing tasks. Indications for such building blocks have been reported in, for example, driving (Shinar et al., 1998), typing (Viviani & Laissard, 1996; Yamaguchi et al., 2012), video gaming (Thompson et al., 2017), and building LEGO walls (Arnold et al., 2017). These building blocks consist of representations of cognitive and (perceptuo-) motor skills so that these skills require little attention to execute (Fitts, 1964; Newell & Rosenbloom, 1981). These reduced attentional demands can explain that practice is not only characterized by faster and more fluent performance but at the same time that skilled performers behave more responsively to changing sensory inputs (MacKay, 1982). Aligned with enhanced processing efficiency, skill development frequently coincides with a shift and decrease of neural activity in the brain (Chein & Schneider, 2005; Picard et al., 2013; Verwey et al., 2019).

A recent literature review attributed the development of high-level motor skills to improved selection of movement goals and of actions along with an improved skill to execute those actions (Krakauer et al., 2019). Skilled action execution, in turn, has been argued to involve learning arbitrary visuomotor mappings and learning to execute movement sequences (Doyon et al., 2003). Learning visuomotor mappings allows us to compensate for, and adjust to, environmental changes and to skillfully use hand tools. This is investigated in motor adaptation studies ( e.g., Redding & Wallace, 2002; Rieger et al., 2008; Verwey & Heuer, 2007). Motor sequence learning involves the incremental acquisition of movements into integrated serial behavior, and this is the topic of the present article. It presents a review of studies that used the Discrete Sequence Production (DSP) task (Verwey, 1994b, 1999) and proposes a novel version of an earlier published processing framework (Verwey et al., 2015).

The initial inspiration to develop the DSP task came from the interest to better understand why the development of car driving skill is accompanied by a reduction in cognitive workload (e.g., Shinar et al., 1998; Verwey, 2000; Verwey & Veltman, 1996). In those days, research with the serial reaction time (SRT) task was just getting up steam (Nissen & Bullemer, 1987; Willingham et al., 1989; for reviews, see Abrahamse et al., 2010; Keele et al., 2003). Yet I felt that continuously cycling through a single series of key presses does not account for the development of the short, almost automatic, action series responsible for the car-driving skill (Michon, 1985). The DSP task that was developed to study skilled execution of short motor sequences typically consists of two fixed series of 6 or 7 key presses that are repeated over and over in a random order (Abrahamse et al., 2013; Verwey, 1999). Performing that task initially involves reacting to two series of 6 or 7 so-called key-specific stimuli, but eventually participants learn to rapidly execute the keying sequences as integrated motor patterns—building blocks—that take little attention. Given my interest in skilled motor behavior the DSP task usually involves substantial practice, like 500 trials per sequence. Typical phenomena observed in those DSP sequences are the rapidly increasing execution rate with practice of responses after the first with sometimes interresponse times of less than 100 ms, the tendency to break up longer sequences in successive segments of about 4 key presses, the substantial reliance on key-specific stimuli after practice, and the lack of full awareness of the keying order in many participants. As noted by some researchers pressing a key clearly differs from the typical aiming and reaching movements in most real-world skills. However, using short-duration key presses does provide the possibility to unveil the underlying processes and, as this article attests to, the DSP task has become a fruitful paradigm to explore the processing mechanisms responsible for the development of serial motor skills.

So, the purpose of the present review is unveiling the processes responsible for the development of serial motor skills. Given the complexity and the impressive amount of behavioral data collected over the past, say, 60 years this is done by focusing on the results obtained with the DSP task. Following the Introduction in this section, “The Discrete Sequence Production (DSP) task” section presents an overview of the DSP task and its many spin-offs in the literature. In the section titled “Reviewing DSP task results,” I review the more than 25 years of research reported in over 100 DSP and DSP-like studies. This review then culminates in the section titled “C-SMB 2.0” in proposing the second version of the Cognitive Framework of Sequential Motor Behavior or C-SMB 2.0, which succeeds the earlier C-SMB framework proposed in Verwey et al. (2015). That section ends with notions on ways to assess the validity of C-SMB 2.0 and speculations on the neural basis of C-SMB 2.0. In order to put C-SMB 2.0 into a broader perspective, section “Related behavioral paradigms” discusses various related models of motor sequence learning. Conclusions and final comments are presented in the final section. This article ends with a glossary of the terms associated with C-SMB 2.0.

## The Discrete Sequence Production (DSP) task

This section documents the many variants of the DSP task in order to aid future researchers in designing DSP studies. The results obtained with these variants are reviewed in the section titled “Reviewing DSP task results.”

### Defining properties and typical findings

**Fig. 1:**
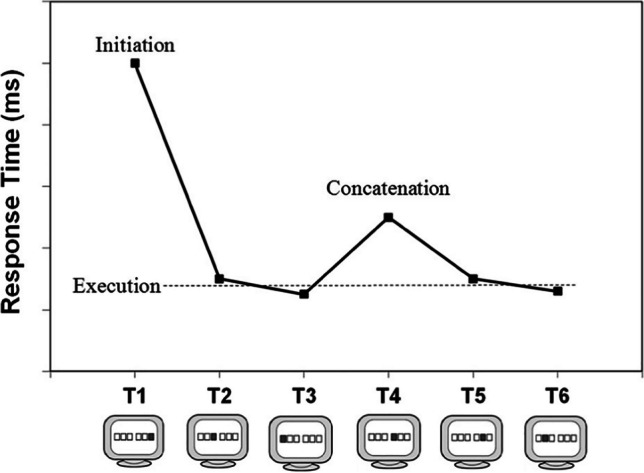
Results typically obtained with a 6-key DSP sequence after 500 practice trials. RT (or T_1_) is assumed to involve sequence initiation, T_4_ reflects concatenation of segments, and the remaining responses are execution responses (copyright granted by Abrahamse et al., 2013)

The 6 or 7 key-specific stimuli indicating as many individual key presses are usually denoted as S_1_ through S_n_. Each of these stimuli consists of filling with a color one of 3–9 predisplayed squares called placeholders. Each stimulus is then responded to by the spatially compatible key press at sequence Positions 1 through *n*, which are designated R_1_–R_n_. Following depression of a key, the next stimulus is displayed, usually after a response–stimulus interval (RSI) of 0 ms^1^. In DSP sequences, a distinction is made between the initiation interval, the execution intervals, and the concatenation interval that separates successive segments (Fig. 1). Care is usually taken that successive keys, like D F and G on a regular QWERTY keyboard, cannot be pressed by rotating the forearm (Rose, 1988, prevented this with an adjustable wrist strap). Depending on the number of keys and sequence length, individual key presses occur equally often in each sequence, and a key press is never immediately repeated. As finger-specific effects have been observed in choice-RT studies (Adam, 2008; Adam et al., 2006; Leuthold & Schröter, 2011; but see Adam & Van Veggel, 1991; Welford, 1971), in DSP sequences finger-specific effects are counteracted by balancing across participants the fingers over the serial positions.

The typical number of around 500 practice trials per sequence constitutes a compromise between experimental convenience with practice taking just a few hours and still developing a sequential motor skill. Response times as short as 100 ms (Verwey, 1996; Wymbs & Grafton, 2015) suggest that after practice these responses are no longer based on reacting to the preceding key-specific stimulus.^2^ Sequence completion is usually followed by an empty screen for 1,000 ms, after which empty placeholders are displayed for another 1,000 ms. This is followed by the first stimulus, S_1_, which consists of filling one placeholder. At the end of practice, participants perform in a test phase, in which specific task variables are manipulated. Counterbalancing sequences across practice and testing ensures that across participants familiar and unfamiliar sequences comprise the same sequence sets. Multiday practice and testing on a day after practice allow for studying consolidation of sequencing skill (Kim et al., 2016; Verwey et al., 2022a, b; Wright et al., 2010). To determine the contribution of explicit knowledge, the practice or test phase in a DSP task is usually followed by an awareness test (see section “Preparing Longer Sequences”).

### Variations of the DSP task

The name Discrete Sequence Production, or DSP, task was coined by Verwey (1996), and the task got its standard form in Verwey (1999). It was developed to study short action sequences. In addition to unveiling the underlying processing mechanisms, the DSP task has been used to address the effectiveness of procedures to boost motor sequence learning, like using mental practice (Sobierajewicz et al., 2016), and increasing contextual interference by randomly varying alternative sequences in a single block of trials (Random Practice, or RP), instead of practicing different sequences in separate blocks (Blocked Practice, or BP)^3^ (Cross et al., 2007; Immink & Wright, 1998; Kim et al., 2018; Lin et al., 2012, 2016; Verwey et al., 2022b). In addition, DSP studies explored the learning benefits of noninvasive stimulation of brain areas like M1 and prefrontal areas using transcranial magnetic brain stimulation (TMS; Cohen et al., 2009; Kennerley et al., 2004; Ruitenberg et al., 2014; Verwey et al., 2022a) and transcranial direct current stimulation (tDCS; Greeley et al., 2020, 2022; Kim et al., 2020, 2021; Kim & Wright, 2020; Sobierajewicz et al., 2019; Waters-Metenier et al., 2014).

The simplicity of the DSP task allowed studies aimed at uncovering the neural substrate of motor sequencing with MEG (Kornysheva et al., 2019), EEG (De Kleine & Van der Lubbe, 2011; Schröter & Leuthold, 2009; Sobierajewicz et al., 2016; Van der Lubbe et al., 2021), and fMRI (Bassett et al., 2015; Jouen et al., 2013; Kornysheva & Diedrichsen, 2014; Lin et al., 2011; Verwey et al., 2019; Wiestler & Diedrichsen, 2013; Wiestler et al., 2014; Wymbs et al., 2012; Wymbs & Grafton, 2009, 2013, 2015; Yokoi et al., 2018; Yokoi & Diedrichsen, 2019).

#### Versions of the DSP task

A number of studies by various research groups reported using DSP tasks even though they sometimes bore other names, including, quite confusingly, the name serial reaction time (SRT) task (e.g., Brown & Carr, 1989; Immink & Wright, 1998; Kornysheva & Diedrichsen, 2014; Kornysheva et al., 2013; Lin et al., 2011, 2012, 2018; Perlman et al., 2010; Schneider & Eberts, 1980 as cited and described in Schneider & Fisk, 1983). These DSP studies are considered here, too, though they often differ from the typical DSP task in terms of stimuli, sequence lengths, amounts of practice, imposed segmentation, timing and rhythms, and/or preparation times.

Two DSP studies involved placeholder fillings with the same luminance as the background to reduce attentional capture (Riesenbeck, 2021; Verwey, 2021). Stimulus locations were not always spatially compatible with the required responses (Ganor-Stern et al., 2013; Verwey et al., 2020) and many DSP tasks involved letters or numbers to indicate the individual responses and/or fingers to be used (e.g., Brown & Carr, 1989; De Kleine & Verwey, 2009; Ganor-Stern et al., 2013; Immink & Wright, 1998; Kornysheva et al., 2013; Mantziara et al., 2021; Verwey, 1999, 2001; Verwey et al., 2002, 2016, 2022a; Wiestler & Diedrichsen, 2013; Wright et al., 1996; Yokoi et al., 2017; Yokoi & Diedrichsen, 2019). These key-specific stimuli have sometimes been displayed simultaneously, after which the sequence was either immediately executed or only after onset of a ‘go’ stimulus (Schneider & Eberts, 1980 as cited and described in Schneider & Fisk, 1983; Wiestler & Diedrichsen, 2013; Wiestler et al., 2014; Wright & Shea, 1991, 1994; Wright et al., 1996). These stimuli have also been first displayed in succession for 750 ms each before the go stimulus in a go/no-go paradigm marked sequence initiation (De Kleine & Van der Lubbe, 2011; Ruitenberg et al., 2012a, b, 2015; Sobierajewicz et al., 2016, 2017a, b; Verwey, 1996; Wymbs & Grafton, 2015). In one study, execution rate of 7-key sequences was about 150 ms slower immediately after learning letter series than when reacting to key-specific stimuli, but already after 160 practice trials this performance difference had vanished (De Kleine & Verwey, 2009).

Several DSP studies investigated effects of sequence preparation by having participants first perform an unrelated choice-reaction or random sequencing task after which a DSP sequence was to be immediately executed (Verwey, 1999, 2001, 2003b; Verwey & Eikelboom, 2003; Verwey & Wright, 2004). Preparation of short, timed sequences was investigated in the dit-dah task that includes 4-element sequences with a predefined timing (Klapp, 1995, 2003). This task has also been studied in a self-select version that assessed the time participants take to prepare a sequence before initiating it (Immink & Wright, 1998, 2001; Magnuson et al., 2004, 2008; Wright et al., 2004). Other studies manipulated preparation time by using the timed response procedure (see Hening et al., 1988; Verwey & Heuer, 2007) which involves a go stimulus after an interval that is often too short to allow full preparation (Ariani & Diedrichsen, 2019; Stöcker & Hoffmann, 2004).

Several DSP task studies used other numbers of responses than the usual 6 or 7. Some involved shorter sequences (1- and 3-key sequences in T. L. Brown & Carr, 1989; Verwey, 1999; 2-key sequences in Verwey, 2001, 2003a; 5-key sequences in Verwey & Wright, 2004; Wiestler et al., 2014). Others involved longer sequences (10-key sequences in Wymbs & Grafton, 2015; 11-key sequences in Yokoi & Diedrichsen, 2019; 12-key sequences in Bo & Seidler, 2009; Kennerley et al., 2004; Wymbs et al., 2012). This allowed studying in detail the development of spontaneous segmentation, but even with 6- and 7-key sequences participants appeared to spontaneously execute the sequences in segments differing across participants (Verwey, 2003a; Verwey et al., 2009; Verwey & Eikelboom, 2003). Vice versa, some studies showed identical segmentation patterns across participants despite the counterbalancing of keys across sequence positions. This was observed when 6- and 7-key sequences were executed with specific response orders (De Kleine & Verwey, 2009; Ruitenberg et al., 2012a, b; Verwey, 2021; Verwey et al., 2014, used one specific basic sequence to impose the same segmentation across participants; Verwey & Dronkers, 2019; Verwey et al., 2022b, used another).

While most DSP sequence are *unstructured* in that they do not impose pauses at fixed sequence positions, a number of DSP studies did impose a segmentation pattern of 6- and 7-key sequences by having participants practice *prestructured sequences* with a pause randomly varying between about 300 to 2,000 ms at one or two fixed sequence positions (Ruitenberg et al., 2015; Verwey et al., 2014, 2019, 2020). Two studies compared the results of practicing with different segmentation patterns for the same sequences (Verwey et al., 2009, 2010)while another study compared spontaneous and imposed segmentation for different groups practicing the same sequences (Verwey, 2021). Segmentation has also been imposed by instruction (Popp et al., 2020). Several studies involved cycling through a fixed series of key presses, but unlike the SRT task in which participants cycle through a series of about 12 stimuli without any breaks, segmentation was stimulated by pauses at specific positions (Ganor-Stern et al., 2013; Stadler, 1993, Experiment 2; Verwey, 1996; Verwey & Dronkert, 1996). A few other studies involved discrete sequences consisting of 4 or 5 cycles of the same 5-key sequence (Wiestler et al., 2014; Yokoi et al., 2017). Several DSP studies imposed complicated timing patterns to examine rhythm learning (Bengtsson et al., 2004; Kornysheva & Diedrichsen, 2014; Kornysheva et al., 2013; Mantziara et al., 2021; Ullén & Bengtsson, 2003).

DSP studies usually end with a test phase in which variations of practiced sequences are compared with control sequences. These controls consist of *unfamiliar sequences* or *random sequences* that consist of discrete series of random responses (e.g., Barnhoorn, et al., 2019a, b; Verwey & Wright, 2014; Verwey et al., 2022b). While the random sequences involve successive reactions to key-specific stimuli, unfamiliar sequences already show some sequence learning within the test block. The ability to generate practiced sequences without guidance by key-specific stimuli is investigated in the *single-stimulus condition*, in which the practiced sequences are to be produced in response to just the first key-specific stimulus or to a general, sequence-specific stimulus (Barnhoorn et al., 2019a, b; De Kleine & Verwey, 2009; Mantziara et al., 2021; Rose, 1988; Ruitenberg et al., 2014; Verwey, 1999, 2010; Verwey et al., 2011, 2022b).

While the DSP task usually includes about 500 practice trials per sequence, practice has been varied between 18 trials (Cross et al., 2007; Immink & Wright, 1998) and 1060, 2000, 2070, and 2310 practice trials per sequence (Verwey & Wright, 2004; Acuna et al., 2014; Wymbs & Grafton, 2015; Verwey, 1996, respectively). And studies using animals like rhesus monkeys (Acuna et al., 2014; Hikosaka et al., 1999; Jin & Costa, 2015; Picard et al., 2013; Ramkumar et al., 2016; Terrace, 2001), rats (Fountain, 1990; Fountain et al., 2007; Macuda & Roberts, 1995; Terrace, 2001), and pigeons (Terrace, 1991, 2001) involved months and years of practice and included over 100,000 practice trials of 8- and 12-element keying sequences (Desmurget & Turner, 2010; Matsuzaka et al., 2007). The number of practice trials is not trivial because sequence learning is assumed to occur at the motor level only after hundreds of practice trials (Verwey & Wright, 2004) and learning may continue even when performance no longer improves (i.e., overlearning; Soderstrom & Bjork, 2015). The test phase in the more typical DSP studies include between 12 (Ruitenberg et al., 2013; Verwey et al., 2022b) and 40 trials per sequence (Verwey, 2021). Wright and Shea (1991) even used only 1 test trial per sequence.

The merits of various practice regimes on skill development have been explored with the DSP task too. Some studies involved blocking and mixing different DSP sequences to assess the effect of contextual interference after limited (Cross et al., 2007; Immink & Wright, 1998; Verwey et al., 2022b) and extended practice (Verwey et al., 2022b). Another study assessed the effect on learning of displaying key-specific stimuli only when no response was given within 800 ms (Verwey, 2021). A few studies enforced the lasting use of key-specific stimuli during practice by displaying a deviating stimulus at an unpredictable sequence position (Verwey, 2015; Verwey & Abrahamse, 2012; Verwey & Wright, 2014). Other studies examined the effect on sequence learning of imposing a very low execution rate during practice (Verwey & Dronkers, 2019) and of instructing participants to be either very accurate or very fast (Barnhoorn et al., 2019a, b).

The attentional demands of initiating and executing DSP sequences have been explored with secondary tasks. These involved remembering series of numbers displayed in advance (T. L. Brown & Carr, 1989; Verwey, 2003a) and counting the number of target tones presented during sequence execution (Verwey, 1993; Verwey et al., 2010, 2014). The latter paradigm gave more robust interference with sequence execution than the first one, possibly because remembering numbers allows participants to refrain from repeating verbal short-term memory (STM) contents for a few seconds without losing the STM content (e.g., Baddeley, 2003).^4^

In DSP tasks usually fingers of one or both hands depress their own key. After some practice, one-handed keying appears to become faster than when fingers of two hands are used (Jiménez, 2008; Maslovat et al., 2016; Verwey et al., 2009). Most DSP studies only assessed key depression so that the next key can be depressed before the preceding key has been released. Still, two studies did require key release before pressing the next key (Schröter & Leuthold, 2008, 2009). A few studies involved aimed movements with one or two adjacent fingers hitting successive keys (Bengtsson et al., 2004; Brown & Carr, 1989; Ullén & Bengtsson, 2003; Verwey, 1993, 1994a, 1995). This was also the response method in the animal studies because animals like monkeys and rats cannot independently move their fingers very well (Desmurget & Turner, 2010; Fountain, 1990; Fountain et al., 2007; Macuda & Roberts, 1995; Matsuzaka et al., 2007). After 280 practice trials, using one finger to depress successive keys appeared to slow the initiation, but not the execution, of 5-keys sequences relative to when four fingers of the left hand were used (Sobierajewicz et al., 2017a, b). Other versions of DSP-like tasks involved forearm movements in a flexion-extension task (Barnhoorn et al., 2016; Shea & Kovacs, 2013; Shea et al., 2011) and a dance-step version involving stepping goal locations with both feet (Chan et al., 2022). Finally, a few fMRI studies used isometric key press movements instead of depressing keys (Kornysheva & Diedrichsen, 2014; Wiestler et al., 2014; Yokoi et al., 2017; Yokoi & Diedrichsen, 2019).

#### Identifying segments

Verwey (2001) distinguished five potential performance features to determine the transition between successive sequences and concluded that this was reflected best by relatively slow responses. The RT difference between slow and fast responses to determine concatenation or segmentation of longer sequences has been statistically tested with planned comparisons (in most studies by Verwey), paired *t* tests (Bo et al., 2009; Bo & Seidler, 2009; Kennerley et al., 2004; Ruitenberg et al., 2012a, b; Ruitenberg et al., 2013, 2014, 2015), and whether a threshold such as 1 standard deviation is exceeded (Scarf et al., 2018; Verwey & Eikelboom, 2003). More sophisticated methods included *k*-means clustering (Song & Cohen, 2014), dynamic network analyses (Wymbs et al., 2012), nonparametric rank-order algorithms (Alamia et al., 2016), and hidden Markov models (Acuna et al., 2014). Interestingly, the procedure proposed by Acuna et al. (2014) distinguishes concatenation on the basis of response times, errors, and their correlations. That study confirmed that response times are powerful indicators for segmentation in the first hundreds of practice trials, but after about 2,000 practice trials—when concatenation responses become fast too—errors and correlational information constitute an increasingly important segmentation indicator. Most DSP sequences showed substantial individual segmentation differences and in one study this was even observed with prestructured sequences (Ruitenberg et al., 2014). These individual differences may have been caused by the counterbalancing across participants of fingers over sequence positions.

## Reviewing DSP task results

This section offers an elaborate review of the findings with the large variety of DSP studies. The structure of this review is based on the assumptions of C-SMB 2.0 as presented in section titled “C-SMB 2.0.” In brief, C-SMB 2.0 assumes that processes are carried out by modality-specific processors at the perception and motor ends of the cognitive system, called perceptual processers (PPs) and motor processors (MPs), with the central processor (CP) in between. Sequence learning occurs in two ways. Repeated preparation of up to about 4 abstract response representations in STM yields so-called *central-symbolic representations*. Concurrently, associative learning occurs at each of the processing levels, perceptual, central, and motor. In addition, participants may develop and use verbalizable, explicit sequence knowledge and they develop additional skills that benefit the execution of DSP sequences in general.

The section titled “The role of key-specific stimuli” presents indications for the lasting reliance on key-specific stimuli and how this can be reduced. Indications for the rapid development of central-symbolic representations in STM are discussed in “Central-symbolic sequence representations.” Results showing the slow development of associative sequence representations at various processing levels, and how the CP and the MP use these representations, are reviewed in section “Associative learning.” Section “Explicit sequence knowledge” discusses findings regarding the development of explicit sequence knowledge, how that knowledge contributes to the execution and segmentation of DSP sequences, and why more aware participants are faster after moderate but not after extended practice. Next, “General skills” examines evidence that practicing DSP sequences also yields general skills that benefit the execution of any DSP sequence. Finally, a few indications from DSP studies are considered in section “Executive control” for the possibility that the control and learning of response sequences may involve similar mechanisms as the executive control of successive processes. Each of these sections starts by highlighting the section’s main findings, which is then followed by reviewing the relevant studies and the section’s main conclusions.

### The role of key-specific stimuli

To account for the results of the DSP task, we earlier proposed the Dual Processor Model (DPM; Abrahamse et al., 2013; Verwey, 2001) and the Cognitive framework of Sequential Motor Behavior (C-SMBl Abrahamse et al., 2013; Verwey et al., 2015). These models suggested that after substantial practice, the second and later stimuli indicating which key to press are no longer used once the first stimulus has been identified. Indeed, no longer displaying these key-specific stimuli after practice slowed sequence execution by 155 ms per response after 144 practice trials (Verwey et al., 2011) and by only 32 ms after 720 practice trials (Ruitenberg et al., 2014). Nevertheless, removing key-specific stimuli after the typical 500 practice trials with key-specific stimuli made it impossible for about a third of the participants to produce the sequences they had been practicing (Ruitenberg et al., 2014; Verwey, 1999; Verwey et al., 2011). And in a cycling version of the DSP task, which involved continuous repetition of a 9-key sequence with pauses at fixed sequence positions, about a third of the participants still showed no segmentation after practice suggesting they continued reacting to the key-specific stimuli (Verwey, 1996). That key-specific stimuli continue to be used in DSP sequences was demonstrated also by participants not being able to ignore key-specific stimuli when these became harmful (Verwey et al., 2020). This suggested that the luminance change relative to the background of the key-specific stimuli attracts visual attention (Jonides & Yantis, 1988; Yantis & Jonides, 1984) and the resulting attention shift then primes the spatially compatible response (Rubichi et al., 1997; Van der Lubbe et al., 2012). In these DSP studies, participants were not informed about the luminance change because the capturing of attention is an automatic process and participants quickly learned this given the high number of DSP trials. The finding that changing the irrelevant stimulus features of general display location and placeholder shape did not affect sequence execution (Ruitenberg, Verweym & Abrahamse, unpublished work in 2010) suggests a role of visual attention here (e.g., Wolfe, 2021). While participants cannot learn to ignore luminance increases in DSP sequences, they are able to learn focusing on relevant stimuli and even just on the distinguishing or rapidly available stimulus feature (Verwey, in press-b), and ignore irrelevant stimuli.

When key-specific stimuli do not involve a luminance change, participants seem able to prepare whether or not their attention is attracted by a color change (Müller et al., 2003). This was suggested by the finding that reliance on occasionally displayed key-specific stimuli reduced when these stimuli were isoluminant, and that this reliance disappeared when participants were fully aware of the sequence (Verwey, 2021). As this study involved effects of only occasionally displayed key-specific stimuli, a recent pilot experiment assessed stimulus dependence when all key-specific stimuli were isoluminant (Riesenbeck, 2021; also see Gaspelin & Luck, 2018; Lambert et al., 2003). This experiment still showed lasting reliance on the stimuli. Yet a follow-up experiment showed that this reliance did not occur when the first key-specific stimulus did involve a luminance change while the ensuing stimuli did not. The lasting need to identify the first stimulus, which was isoluminant, seems to have forced participants in the first experiment to also process later isoluminant stimuli while the later isoluminant stimuli were ignored when the first stimulus was not isoluminant. So, these studies demonstrate that participants remain dependent on key-specific stimuli in typical DSP sequences because the luminance change associated with onset of the key-specific stimuli continues to attract attention.

### Central-symbolic sequence representations

The DSP research in this section on central-symbolic representations suggests that when executing different motor sequences in, say, the first 100 practice trials, the first responses of the various potential sequences are prepared in STM on the basis of explicit, episodic knowledge. Development of S-R_1_ associations with practice then allows immediate execution by the MP of the first response once the first stimulus has been identified. While that first response is being executed the 3 or 4 ensuing responses are prepared in STM too. The repeated preparation of the same series of responses in STM during practice is assumed to prompt the gradual development of a multimodal central-symbolic representation which consolidates after practice has ended. In simple-RT (i.e., blocked) tasks display of the go stimulus is after some practice preceded by the concrete preparation at the motor level of the motor features making up the first response, and by the preparation of the abstract response representations of the ensuing 3 or 4 responses in STM. This allows immediate and rapid execution of the first response upon display of the go stimulus. The need to always produce the same sequence in simple-RT conditions implies that responses are activated in STM only once, at the start of a trial block. Consequently, central-symbolic representations develop slowly in simple-RT conditions.

Central-symbolic representations code movement sequences in STM as a task-dependent mixture of various spatial, and sometimes also verbal, response representations. Even sensory feedback is probably included in this representation (cf. the Theory of Event Coding, or TEC; Hommel et al., 2001). This mixture changes in the course of practice. In the case of longer sequences, responses after the first segment are initially produced in the so-called reaction mode, which depends on reacting to each key-specific stimulus in a DSP sequence, but after some practice they too are first prepared in STM. This prompts development of another central-symbolic representation. This particular later central-symbolic representation is initially selected by the CP while the MP is executing the preceding segment. In the case of the last segment the CP is free to concurrently select the last response. The fact that a segment or response representation can be selected during execution of the preceding segment implies that that knowledge is initially stored in another STM component than the individual responses of that preceding segment (Baddeley, 2000). With further practice this abstract central-symbolic representation of the later segment is automatically triggered through associations with the preceding segment and its responses. The fact that the concatenation response is relatively slow indicates that preparing a central-symbolic representation by specifying the constituting abstract response representations in STM can occur only when the last response in that component of STM has been executed and it becomes available again. The reliance on key-specific stimuli can be reduced when practice allows participants to ignore key-specific stimuli—for example, by using single-stimulus practice, mental practice, or displaying isoluminant stimuli after the first, sequence-specific stimulus. These insights are based on the review in the sections below.

#### Short-term memory

Earlier models, including C-SMB, postulated that preparation of short movement sequences occurs in a short-term, limited-capacity motor buffer (Abrahamse et al., 2013; Rosenbaum et al., 1983; Sternberg et al., 1978; Verwey et al., 2015). DSP studies, however, suggest that it is in STM that the preparation of the up to about 4 abstract response representations occurs (Baddeley, 1986; Baddeley & Hitch, 1974; Klapp, 1995, 2003; Logie & Cowan, 2015). One indication for the use of STM is that participants can execute motor sequences on the basis of verbal letter and number series they earlier memorized (Brown & Carr, 1989; De Kleine & Verwey, 2009; Kornysheva et al., 2013; Mantziara et al., 2021; Ruitenberg et al., 2014; Verwey, 1999, 2001; Verwey et al., 2002, 2016, 2022a; Wiestler & Diedrichsen, 2013; Yokoi et al., 2017; Yokoi & Diedrichsen, 2019). Another indication that preparation actually occurs in STM is that individuals with a higher STM capacity spontaneously develop longer segments in DSP sequences (Barnhoorn et al., 2016; Bo et al., 2009; Bo & Seidler, 2009; Seidler et al., 2012).

A further indication that STM is indeed involved in generating DSP sequences is that several phenomena in the typical STM task of short-term verbal list learning can be observed with DSP sequences too. That is, the 4-item limit known from serial letter and number learning (Baddeley, 2003; Cowan, 2000; Logie & Cowan, 2015) also delimits the DSP segments (Acuna et al., 2014; Ariani & Diedrichsen, 2019; Bo & Seidler, 2009; De Kleine & Van der Lubbe, 2011; Ganor-Stern et al., 2013; Greeley et al., 2020; Ruitenberg et al., 2012a, b; Sobierajewicz et al., 2016, 2017a, b ; Verwey, 1996). And longer sequences in both verbal list learning (Cowan, 2000) and in DSP sequence learning (e.g., Bo & Seidler, 2009; Verwey & Eikelboom, 2003) are segmented in similar ways (like in data entry task; Fendrich & Arengo, 2004). Furthermore, the primacy and recency effects known from verbal list learning and recognition memory (Ebbinghaus, 1885; Johnson, 1991; Wright et al., 1990) emerge in DSP sequences as higher awareness and execution performance of the first and last responses (De Kleine & Van der Lubbe, 2011; De Kleine & Verwey, 2009; Verwey et al., 2009, 2010; Verwey & Wright, 2014).

Another sign that DSP sequences are prepared in STM comes from evidence that sequence representations include nonmotor information. This is demonstrated by go/no-go DSP studies, in which the key-specific stimuli are all displayed before sequence execution. When after practicing DSP sequences, in these studies irrelevant features of the key-specific stimuli like their color, shape, and general display location were changed to those of another practiced DSP sequence, execution of the sequences was slowed, even though the stimuli were not visible any more at sequence initiation (Ruitenberg et al., 2012a, b; Wright & Shea, 1991, 1994). The fact that this slowing was larger with 4- than with 3-key sequences (Wright & Shea, 1991, 1994) confirms that 4-key sequences fully loaded STM. That this slowing was stronger after 50 than 250 practice trials (Ruitenberg et al., 2012a, b) is consistent with the assumption that associative sequence learning gradually accompanies control by the STM-based central-symbolic representations (see below). However, after 500 practice trials STM still seems involved. This is indicated by the slowed initiation of other sequences and a doubling of the number of execution errors when STM load was increased by reversing stimulus–sequence mapping (Verwey, 1999).

These behavioral indications that DSP sequences are prepared in STM are complemented by neurophysiological findings. Reduced strength of the CNV and CDA components of the EEG after 84 practice trials with each of eight 6-key sequence, relative to unfamiliar sequences, showed greater involvement of visual STM in preparing unfamiliar than familiar sequences in a go/no-go DSP study (De Kleine & Van der Lubbe, 2011). A follow-up study showed enhanced activity on the occipital electrodes during preparation of sequences that had received little or no physical practice (Sobierajewicz et al., 2016). This too was taken as evidence for involvement of visual STM. Additionally, after 30 and also after 212 practice trials per sequence, the preparation of 3- to 5-element motor sequences (Averbeck et al., 2002; Kornysheva et al., 2019) was associated with activation of the neural areas with an established role in STM, the prefrontal cortex (BA46), and the parahippocampal region. All in all, there are many indications that preparation of discrete motor sequences actually occurs in STM rather than in a short-term motor buffer.

#### Preparation at the motor level

In addition to preparing up to about four abstract response representations in STM, this section reports indications that the first response can also be prepared in great detail at the motor level. In retrospect, indications for at least two response preparation levels have been reported by a variety of studies. Support for this notion comes from an in-depth analyses of RT distributions in a choice-RT task (Meyer et al., 1985), from the divergence of behavioral and psychophysiological measures of response preparation (Miller et al., 1996), and from the well-known distinction between abstract and muscle-specific programming (e.g., Klapp, 1977; Sternberg et al., 1978). Preparation at different processing levels is consistent also with additive factors analyses showing different processing stages for sequence selection and executing individual sequence elements (Sanders, 1990; Verwey, 1999; Verwey et al., 2015), and with Sternberg et al.’s (1978) distinction between retrieval of abstract response representations from a buffer and unpacking the retrieved representation to derive the individual motor elements.

Advanced preparation at the motor level probably involves the construction of an executable response representation consisting of motor features like the performing hand, movement direction and extent (cf. parameter specification in schema theory; Schmidt, 1975; Shea & Wulf, 2005). These response features can be prepared in any order and at different moments (Leuthold & Jentzsch, 2011; Rosenbaum, 1980, 1983; cf. Miller, 1993). Individual response features can be prepared before display of the imperative stimulus, and once specified, they remain active and can be reused with later responses (Rosenbaum et al., 1986, 2007).

Two fMRI studies corroborate that in a simple-RT condition the first response of DSP sequences is prepared at an advanced, motor level. One showed advanced preparation of the finger giving the first response of a motor sequence in a simple-RT condition by way of increased activation of the sensorimotor cortex (S1/M1; Yokoi et al., 2018; Yokoi & Diedrichsen, 2019). A network of secondary motor areas (like dorsal premotor cortex-PMd, supplementary motor area-SMA, and the posterior parietal cortex-PPC) seemed to represent the ensuing responses in a more abstract code. Also, EEG studies showed by way of the LRP component that the hand executing the first response in a DSP sequence was activated almost immediately, irrespective of the length of the motor sequence (Schröter & Leuthold, 2008, 2009; Smulders et al., 1995). The fact that sequence initiation time in those studies still showed the longer initiation time as the number of responses in the sequence increased (i.e., the *sequence length effect*) suggested that other features of the first response were prepared at the motor level only later, after all other responses had been prepared in an abstract form in STM. So, there is considerable evidence for the notion that the first response can be prepared in detail at a motoric processing level.

#### Shorter DSP sequences

The sequence length effect constitutes the slower initiation of sequences with up to 4 or 5 elements as the sequence gets longer (Henry & Rogers, 1960; Klapp, 2003; Schröter & Leuthold, 2008; Sternberg et al., 1978; Verwey, 1999). The sequence length effect is generally assumed to occur in simple-RT conditions, in which the same sequence is repeated in a block of trials, where it reduces and disappears with practice. It is usually not observed in choice-RT conditions, in which 2 or 3 sequences are to be executed in an unpredictable order (e.g., Klapp, 2003; Verwey, 1999). In both conditions, absence of the sequence length effect has been attributed to participants immediately executing the first response upon stimulus display (Klapp, 2003; Portier et al., 1990).

In simple-RT conditions, the responses of short sequences are basically prepared in STM at the start of a block of trials (Averbeck et al., 2002; Immink & Wright, 1998; Kornysheva et al., 2019; Sternberg et al., 1978; Wright et al., 2004), in the case of unfamiliar sequences probably in the order in which they are executed (Ulrich et al., 1990). The sequence length effect in simple-RT conditions has been attributed to the longer time it takes to search STM for the first response (Rosenbaum et al., 1983; Sternberg et al., 1978). Competitive queuing models attribute the sequence length effect to the greater competition as there are more sequence elements (Bullock, 2004; Burgess & Hitch, 1999; Kornysheva et al., 2019). Participants can be instructed to immediately execute the first response (Sidaway, 1994) but with practice they appear to do this any way. I propose to attribute the resulting reduction of the sequence length effect with practice to the first response eventually being prepared at the motor level of processing before display of the imperative stimulus. Displaying the imperative stimulus then immediately triggers, also depending on task- and goal-dependent S-R_1_ associations, R_1_ in a reflex-like way (Hazeltine & Schumacher, 2016; Hommel, 2000). In support of enhanced preparation reducing the sequence length effect, this effect returned when after practice in a simple-RT task preparation was hampered by first executing another sequence (Verwey, 1999).

In choice-RT conditions, absence of the sequence length effect can be attributed to the concurrent preparation of the first responses of the alternative sequences in STM before display of the imperative stimulus (Cisek, 2007; Filevich & Haggard, 2013; Gallivan et al., 2016; Rose, 1988; Schröter & Leuthold, 2009). In choice-RT tasks the first response is immediately executed because the stimulus triggers the associated responses already in STM, first via some S-R_1_ mapping rule and later via an S-R_1_ association (Hazeltine & Schumacher, 2016; Hommel, 2000). Support for the existence of such S-R_1_ associations in DSP tasks is that reversing the learned mapping between a sequence-specific stimulus and a familiar sequence slowed sequence initiation but not the ensuing responses (Verwey, 1999). While the MP is then executing R_1_ the CP activates representations of the remaining responses in the sequence in STM. This activation initially involves individual response representations (Immink & Wright, 1998; Sternberg et al., 1978; Wright et al., 2004), but after practice it may concern the entire central-symbolic representation. Once these responses are prepared in STM, and R_1_ has been executed, the remainder of shorter sequences is ready in STM for execution by the MP (Verwey, 1996).

#### Longer DSP sequences

Sequence initiation time in simple-RT conditions does not increase further when sequences exceed the STM limit of about 4 responses (Sternberg et al., 1978). Instead, the *rate effect*—the increase of the mean time between successive responses in longer sequences (Monsell, 1986; Sternberg et al., 1978)—signifies the online preparation of later responses that cannot immediately be activated in STM. Verwey (2003a) demonstrated that in 6-key DSP sequences this rate effect was caused in part by the occurrence of one or sometimes two slow responses halfway through those sequences because the sequences include successive segments. The studies reviewed below further unveil the processes involved in preparing and executing longer sequences.

##### Preparing longer sequences

Within tens of trials the second response of a DSP sequence is often relatively fast, as if the first two responses are initially executed as a unit (go/no-go DSP task: Ariani & Diedrichsen, 2019; De Kleine & Van der Lubbe, 2011; De Kleine & Verwey, 2009; regular DSP tasks: Verwey, 1996, 1999, 2001, 2010, 2015; Verwey et al., 2009, 2010; Verwey & Wright, 2014). That the first few responses are actively prepared in STM is corroborated by the finding that the rapid second response loses its rapidity, especially in 2-key sequences, when preparation is hampered by a secondary memory task (Verwey, 2003a), when another sequence is executed first (Verwey, 2001, 2003b), when changing the practiced stimulus-sequence mapping (Verwey, 2001), and when changing the task from simple-RT to choice-RT (Verwey, 1999, 2003a). With more practice the third and fourth responses become faster too (Ariani & Diedrichsen, 2019; Verwey et al., 2014). This preparation of the initial responses of longer sequences seems to involve use of explicit, perhaps episodic, response knowledge given that more aware participants initiated familiar sequences slower while they executed R_2_ and R_3_ faster after extended practice (Verwey & Abrahamse, 2012; Verwey et al., 2010). However, eventually this involves the application of central-symbolic representations any way.

The execution of still later responses initially relies on responding to key-specific stimuli in the reaction mode. This is indicated by the faster execution of the final 3 or 4 responses in a 6-element DSP sequence in participants with a well-developed reaction skill (Verwey et al., 2014). With more practice, these later responses are integrated in a second central-symbolic representation that can then be selected and activated in STM as a whole too (Kennerley et al., 2004; Popp et al., 2020; Ramkumar et al., 2016; Verstynen et al., 2012; Verwey et al., 2022b; Wymbs et al., 2012).

Several go/no-go DSP studies showed that, when given sufficient preparation time, participants can prepare up to 6 responses (Ariani & Diedrichsen, 2019; De Kleine & Van der Lubbe, 2011; Mantziara et al., 2021; Sobierajewicz et al., 2017a, b). That preparation may exceed the 4-item STM limit when the sequences are practiced when there is ample preparation time, can be attributed to two mechanisms. First, with 5-key sequences the second through fifth responses may be prepared in STM while the first response is prepared at the motor level and not in STM. Second, sequences with 5 and more responses may involve segmentation. This is supported by the slow third response of 5-key sequences after 20 practice trials in a go/no-go task (Sobierajewicz et al., 2017a, b), and by the finding that providing more preparation time in a 5-key go/no-o DSP study increased execution rate of the first three, but not of the later responses (Ariani & Diedrichsen, 2019).

##### Developing segmentation

That central-symbolic representations develop due to the repeated preparation in STM implies that segmentation is affected by the way in which sequences are executed in early practice. Indeed, when a list of stimuli is first presented and learned verbally the sequence can immediately be segmented (De Kleine & Verwey, 2009), thus showing that DSP sequences can be verbally coded in STM (see, e.g., the review by Cowan, 2000). And in a DSP study in which 8 response-specific digits were displayed simultaneously, participants appeared to segment 8-key sequences in response pairs across the first 20 practice trials that were then combined in two successive 4-element segments (Schneider & Eberts, 1980 as cited and described in Schneider & Fisk, 1983). Also, when 6-key DSP sequences were practiced in a go/no-go task in which all key-specific stimuli were successively displayed before sequence initiation, two successive 3-key segments were found already across the first 50 practice trials per sequence (Ruitenberg et al., 2012a, b). In contrast, in regular DSP tasks in which key-specific stimuli guide sequence execution, the relatively slow execution of the fourth and later responses in early practice (Giusti, 2021; Ruitenberg et al., 2012a, b; Verwey & Eikelboom, 2003; Verwey et al., 2022b) suggests that participants first executed the prepared first segment from STM after which they continued by reacting to key-specific stimuli. Also, when participants were cautious while executing familiar sequences because a deviating key-specific stimulus was expected, the first three responses were executed still faster than later responses (Verwey & Wright, 2014) as if the participants initially compared the stimuli with the already prepared responses in STM, after which they truly reacted to the key-specific stimuli.

Another study showed that when a group of participants were reacting to key-specific stimuli the last 3 of 6 responses were slower across the first 40 practice trials, indicating that only the first segment had been prepared (De Kleine & Verwey, 2009). Interestingly, the second segment was executed faster in another group that had first memorized the key-specific letters, indicating that relying on explicit sequence knowledge initially allows faster sequence execution than reacting to key-specific stimuli. Still, in line with development of central-symbolic representations, eventually the segmentation pattern was not different in the two groups. Another study displaying key-specific stimuli during sequence execution showed that even when response stimulus intervals (RSIs) varied between 500 and 2,000 ms, segmentation developed within 30 practice trials (Verwey & Dronkers, 2019). This confirms that segmentation is determined by repeated preparation in STM rather than by actual execution (cf. Mantziara et al., 2021). The limited awareness in this study indicated that the STM content need not be explicit.

##### Determinants of the segmentation pattern

Spontaneously developing segmentation is often concealed by individual differences (Bo & Seidler, 2009; Kennerley et al., 2004; Popp et al., 2020; Verwey, 2003a; Verwey et al., 2009; Verwey & Eikelboom, 2003). Wymbs et al. (2012) showed with 12-key sequences that spontaneous segmentation differed between, and in the course of practice also within, participants. With the detection algorithm they used, these segments included 3.1 responses on average, but this number may well have been affected by the parameters of the segment detection algorithm. With the typical 6- and 7-key DSP sequences the opportunity to develop idiosyncratic segmentation patterns is limited, but even in these sequences there appear several determinants of segmentation. The first is not under direct experimental control and concerns the individual’s STM capacity (Barnhoorn et al., 2016; Bo et al., 2009; Bo & Seidler, 2009). A second determinant is the occurrence of pauses during practice of prestructured sequences (Verwey, 1996; Verwey et al., 2009, 2010, 2014, 2020; though even then not all participants used the imposed segments, Ruitenberg et al., 2015). Segmentation may further be influenced by regularities in element order. We know from older studies with the SRT task and serial pattern learning that segmentation is affected by runs, trills and reversals (Koch & Hoffmann, 2000a; Restle, 1970; Simon, 1972). Still, in the DSP task two particular 7-key sequence structures have been found to prompt the same segmentation across participants despite the counterbalancing of fingers across participants (De Kleine & Verwey, 2009; Ruitenberg et al., 2012a, b; Verwey, 2021; Verwey et al., 2014 used VNBNVBC and its 4 counterbalanced versions; Verwey & Dronkers, 2019; Verwey et al., 2022b used VCBNCVN and its 4 counterbalanced versions).

Other sequencing studies showed that segmentation may be influenced by instruction (Popp et al., 2020), salient events like stimulus color (Jiménez et al., 2011), segment repetition (Ruitenberg et al., 2015; Verwey et al., 2002, 2022a), difficulty of finger transitions (Popp et al., 2020), left-to-right regularities (Verwey & Eikelboom, 2003), and interhand transitions (Jiménez, 2008; Koch & Hoffmann, 2000a; Verwey et al., 2009, but not in Verwey et al., 2016). Even the occurrence of errors in early practice may affect segmentation later on (Sakai et al., 2003). Still, segmentation promoted by these salient events appeared not always robust. Robust segmentation probably depends on whether the occurrence of regularities and salient elements coincide with STM capacity limitations (Verwey et al., 2016) and the speed of specific finger transitions (Jiménez et al., 2011; Popp et al., 2020). In contrast, DSP studies showed no effects on segmentation of the distance between the fingers used with consecutive movements, hand dominance, finger identity (Kennerley et al., 2004; Verwey et al., 2009), performing a preceding task (Verwey & Eikelboom, 2003), a slow finger (Barnhoorn et al., 2019a, b), and segmentation of simultaneously and earlier practiced sequences (Ruitenberg et al., 2015; Verwey et al., 2009; though Verwey et al., 2010, may still have observed transfer in early practice).

##### Benefits of segmentation

The notion that segmentation develops to deal with the limited-capacity of STM (Gobet et al., 2016; Halford et al., 1998) implies that individual segments are executed at high rates but sequences may be executed more slowly as a whole because of the slow concatenation responses. More explicit segmentation of motor sequences may be beneficial with moderate practice but this benefit seems to disappear with more extended practice (Bo & Seidler, 2009). Indeed, a study with a 9-key cycling DSP sequence showed that the benefit of segmentation on sequence execution time was observed after about 800 practice trials but had vanished after about 1,500 practice trials (Verwey, 1996). And when sixteen 12-element DSP sequences were each practiced for 189 trials no correlation was found between spontaneous segmentation of sequences and their execution rate (Wymbs et al., 2012). Also, after 144 practice trials older participants using less segmentation were not generally slower than those showing more pronounced segmentation (Verwey, 2010; Verwey et al., 2011). Similarly, large individual differences in segmentation of 6-key sequences after 500 practice trials were not associated with differences in overall execution time (Verwey & Eikelboom, 2003). So, it seems that segmentation may benefit execution of sequences with moderate practice, but this benefit disappears with more extensive practice, most likely because associative learning then becomes dominant.

##### Combining familiar sequences

When motor sequences are practiced independently they can later be used as behavioral building blocks that can be combined and adjusted as needed (Flash & Hochner, 2005; MacKay, 1982; Rohrer et al., 2004). Verwey (2001) had participants practice separate 2-, 3-, and 4-key sequences in response to sequence-specific stimuli for 640 trials, after which they responded to two concurrently displayed sequence-specific stimuli by executing the two indicated sequences in rapid succession. The practiced sequences appeared robust in that they continued to be executed separately, even in the case of two successive 2-key sequences that could have been executed as an integrated 4-key sequence. Like with successive segments in a single sequence, the second sequence was prepared during execution of the first sequence. And in line with the notion of a unified CP, slowing of the first sequence did not further increase when selection demands of the second central-symbolic representation were made more complex. Interestingly, the second sequence was initiated faster when it had the same length as the first one. This confirms that sequence length is a motor feature, that can be reused (cf. the parameter remapping effect; Rosenbaum et al., 1986, 2007, and the motor hysteresis effect, Kelso et al., 1994; Schütz & Schack, 2015).

##### Concurrent programming

Whereas later responses in DSP sequences are initially executed slowly because they are given in response to key-specific stimuli, after extended practice execution rate of the final segment is in fact even higher than of earlier segments (Ruitenberg et al., 2012a, b; Verwey, 1996, 2001, 2021; Verwey & Dronkert, 1996; Verwey & Eikelboom, 2003). This was the case even when a 3-key segment was executed twice (Verwey et al., 2002, 2022a). The slower execution of the first segment contributes to the slower mean execution rate of 6-key than 2- and 3-key sequences (Verwey, 1999, 2003a). One reason for the relatively slow execution of nonfinal segments in 6-key sequences is that the CP selects and activates the oncoming segment in LTM while the MP is executing the preceding segment so that the CP cannot race with the MP to trigger individual responses (Verwey, 2001).

The hypothesis that the CP and MP may be racing to trigger individual response representations can account also for the slowed execution of DSP sequences when a secondary task is carried out (indicating a reduced CP contribution; Verwey et al., 2010, 2014), and that slowing by a secondary task reduces with practice because the contributions of the MP and of associative sequence learning increase (Verwey, 1993, 2003a; Verwey et al., 2010). The reduced amount of racing after practice also explains slowed sequence execution when other fingers are used than during practice (due to a reduced contribution of the MP; Verwey et al., 2009; Verwey & Wright, 2004), and when in the so-called single-stimulus condition key-specific stimuli past the first are no longer presented (reducing the contribution of stimulus–response translation by the CP; Ruitenberg et al., 2014; Verwey, 2021; Verwey et al., 2020). The detrimental effect of a preceding task on sequence execution (Verwey, 2003b; Verwey & Eikelboom, 2003) can be attributed to the CP not having prepared the required processing strategy for DSP sequences.

That slowing of the ongoing nonfinal segment was not affected by the length of the ensuing segment shows that selecting the next segment involves a central-symbolic representation that is independent of the number of responses (Verwey, 1996). Instead, the greater slowing of ongoing 2- and 3-element segments than of ongoing 4- and 5-element segments by a next segment indicates that preparation of the upcoming segment requires more time than the execution of short preceding segments provides (Ruitenberg et al., 2012a, b; Verwey, 1996, 2001; Verwey & Dronkert, 1996).

It is actually quite remarkable that the first response of a noninitial segment is always relatively slow even though that segment can be selected during execution of the preceding segment (Verwey, 1995, 2001). This can be taken to indicate that the abstract central-symbolic representations can be selected in advance (in one STM component) while preparing the individual but still abstract response representations in STM (Sternberg et al., 1978) occurs only when (another component of) STM is no longer used for executing the preceding segment.

A phenomenon that has attracted little attention is the high execution rate and accuracy of the last response of DSP sequences. The results of various DSP studies suggest that the fast last response occurs primarily in more slowly executed sequences. It was observed in 6- and 7-key DSP sequences in the first practice block, after 210 practice trials, and with unfamiliar sequences in the test phase (De Kleine & Van der Lubbe, 2011; De Kleine & Verwey, 2009; Ruitenberg et al., 2012a, b; Verwey, 2003b; Verwey et al., 2002, 2009, 2010; Verwey & Wright, 2014). The fast last response was observed also in go/no-go DSP tasks across the first 20 to 48 practice trials when 5-key sequences were produced (Ariani & Diedrichsen, 2019; Sobierajewicz et al., 2017a, b, 2018; Van der Lubbe et al., 2021), and across 84 practice trials with 6-key sequences (De Kleine & Van der Lubbe, 2011).

The fast last responses of DSP sequences were observed when these sequences were executed slowly. They were executed slowly because (1) participants executed the sequences in reaction mode (Verwey, 2010, 2015; Verwey & Abrahamse, 2012; Verwey et al., 2011; Verwey & Wright, 2014), (2) stimuli were expected at incompatible locations (Verwey et al., 2020), (3) participants tried to be very accurate without guiding stimuli (Barnhoorn et al., 2019a, b), (4) participants were older (Barnhoorn et al., 2019a, b), (5) there was a transition between the hands when one hand had moved to the side (Verwey et al., 2016), and (6) after practice the pause was removed from prestructured sequences (Verwey, 2010). Early in practice, even the last two responses are sometimes relatively fast (Ruitenberg et al., 2012a, b; Sobierajewicz et al., 2017a, b; Verwey, 2021; Verwey et al., 2016). Instead, the fast last response does not seem to occur after the typical 500 practice trials with unstructured sequences (Barnhoorn et al., 2019a, b; De Kleine & Verwey, 2009; Mantziara et al., 2021; Ruitenberg et al., 2012a, b; Verwey, 1999, 2003a, 2015; Verwey et al., 2009, 2014). It neither occurred in prestructured sequences, even when these had been practiced for less than 500 practice trials (De Kleine & Verwey, 2009; Verwey, 2010, 2021; Verwey et al., 2009, 2010; Verwey & Dronkers, 2019; Verwey et al., 2002), and in random sequences (Barnhoorn et al., 2019a, b; De Kleine & Verwey, 2009; Verwey & Wright, 2014). Together, these findings indicate that the fast last response occurs only in sequences executed at a moderate rate. The finding that more aware participants showed a faster last response when execution rate was also relatively low suggests that it was due to the CP using explicit knowledge of that last response to race with the MP (Verwey, 2015; Verwey & Wright, 2014).

##### Two different concatenation processes

Several DSP studies together make a strong case that only in prestructured sequences concatenation relies heavily on the CP selecting and activating the next abstract central-symbolic segment representation in LTM by loading it into STM. In unstructured sequences, concatenation seems to more quickly automate with practice and eventually does not seem to require the CP. This can be attributed to the shorter time that precedes concatenation responses in unstructured sequences, as this allows associations to develop between the successive central-symbolic segment representations and their responses (Deffains et al., 2011; Stadler, 1995; Verwey, 1996; Verwey & Dronkers, 2019; Verwey & Dronkert, 1996).

That spontaneously developing concatenation responses in unstructured sequences involve other processes than execution responses is demonstrated by the first response of spontaneously developing segments in unstructured sequences responding differently than execution responses to relocating a performing hand (De Kleine & Verwey, 2009; Verwey et al., 2016). This is confirmed by the greater slowing of concatenation than of execution responses in unstructured sequences after rTMS of the pre-SMA (Kennerley et al., 2004; Ruitenberg et al., 2014), and when, in contrast to healthy controls, severe hypokinetic Parkinson disease patients switched between different 3-key segments in a 6-key sequence (Hayes et al., 1998).

That the CP remains involved in concatenation in prestructured sequences and not in unstructured sequences is corroborated by findings that in prestructured sequences the concatenation response suffered more than the execution responses from various manipulations while this was not the case in unstructured sequences. This occurred (1) in a tone counting condition (even when no tone was presented; Verwey et al., 2010), (2) when after 300 practice trials the irrelevant color of the key-specific stimuli was changed (Ruitenberg et al., 2015), and (3) when a key-specific stimulus was unexpectedly displayed in a single-stimulus condition (Verwey, 2021). Furthermore, (4) when in a prestructured sequence the CP contribution was reduced by displaying key-specific stimuli at locations spatially-incompatible with the responses, the concatenation response was (marginally significantly) more slowed than the execution responses (Verwey et al., 2020). An fMRI study confirmed these behavioral indications that other neural processes are involved in prestructured than unstructured 4-key sequences (Jouen et al., 2013).

#### Coding of central-symbolic representations

Evidence for abstract central-symbolic sequence representations comes from the transfer of practice benefits to motor sequences of which the response modality was changed. That is, sequencing skill transferred from practicing sequences with 4 fingers pressing 4 keys to depressing those keys with a single finger (Sobierajewicz et al., 2017a, b), and also to performing the same sequence with flexion-extension movements of the forearm (Barnhoorn et al., 2016; Shea & Aranda, 2005; see Dean et al., 2008). This transfer of sequencing skill obviously cannot be attributed to sequence knowledge at the motor level. All in all, the review in the two sections below indicates that central-symbolic representations consist of a task-dependent mixture of various spatial, and sometimes also verbal, sequence representations. Sequence knowledge seems coded in terms of, implicit or explicit, trunk- or head-based spatial codes that become more important in the course of hundreds of practice trials and that participants can quickly adjust when the hand orientation is changed. Verbal sequence knowledge is likely to always be explicit and develop rapidly, but the processes required to interpret verbal sequence coding makes verbal codes less suitable for coding DSP sequences so that use of these sequence representations is quickly abandoned, and verbal sequence knowledge may even be forgotten.

##### Spatial sequence knowledge

Spatial representations can involve a variety of allocentric (i.e., world-based) reference frames and ego- (i.e., body-) centric reference frames (i.e., relative to the eye, head, shoulder, trunk, forearm, or hand; Bernier & Grafton, 2010; Heuer & Sangals, 1998; Keulen et al., 2002; Leoné et al., 2015; Liu et al., 2007; McIntyre et al., 1998; Shea et al., 2011; Zacks, 2008). Sequential motor behavior has been argued to rely especially on egocentric reference frames (Willingham, 1998), but people appear flexible transforming spatial representations to other reference frames within less than about 100 ms (Derdikman & Moser, 2010; Zacks, 2008), for instance, by rotation (Georgopoulos, 1986; Leoné et al., 2015; Pellizzer et al., 2009; Shepard & Metzler, 1971).

A few DSP studies confirm that, like in the flexion-extension sequencing tasks (Shea et al., 2016) and the SRT task (Haider et al., 2018), sequencing skill may continue to involve a spatial component, even after hundreds of practice trials, depending on, for example, sequence length and performance feedback (Shea et al., 2016). In one DSP study, 7-key sequences had been practiced for 160 trials with one hand on a keyboard rotated by 90 degrees on one side of the body (De Kleine & Verwey, 2009). The same sequences were then performed with the practiced and the unpracticed hand at the same and the opposite sides of the body. The results suggested that sequence learning had induced hand-specific, trunk-, or head-based spatial sequence representations. Only the concatenation response was unaffected by the hand orientation and location, even when responses were not indicated by key-specific stimuli. Instead, execution with the unpracticed hand appeared based on a representation that was independent of hand orientation, perhaps a verbal representation or a rotated body-based or wrist-based spatial reference frame.

Verwey et al. (2016) had participants practice DSP sequences with two hands for 600 practice trials per sequence on a single keyboard in front of them. Then the right hand was moved to a keyboard at the right of the body that was rotated clockwise by 90 degrees. This slowed execution of the responses following each inter-hand transition for the first 15 trials. The findings suggested that sequence learning involves a spatial central-symbolic representation that codes responses in a cross-hand representation with a trunk- or head-based reference frame that participants could mentally rotate to the orientation of the performing hand within 15 trials (Georgopoulos et al., 1986; Shepard & Metzler, 1971). Developing this cross-hand spatial representation seems to take a fair amount of practice, as these effects were found after 600 and not after 80 practice trials.

Neurophysiological support for the use of visuospatial representations during sequence preparation comes from two go/no-go DSP studies assessing EEG activity on the occipital electrodes (Sobierajewicz et al., 2016), and deriving the CDA and parietal CNV components from the EEG (De Kleine & Van der Lubbe, 2011; Van der Lubbe et al., 2021). The gradual reduction of the CDA component with practice is consistent with a reducing contribution with practice of the spatial central-symbolic representation.

##### Verbal sequence knowledge

Spatial knowledge is better suited for representing motor sequences than verbal knowledge. Still, verbal knowledge allows people to, for instance, key in their ATM PIN number or phone number on an unusual keyboard and to express motor sequences vocally. The possibility to store motor sequences in a verbal code relates to the use of inner speech in the case of complex action plans (Meacham, 1984; Tubau et al., 2007; Vygotsky, 1934/1986).

While spatial sequence knowledge may be explicit as well implicit (Keele et al., 2003), verbal sequence knowledge is by definition explicit. A large number of DSP studies confirm that motor sequences can be executed using just learned key-specific letter and number series (Brown & Carr, 1989; De Kleine & Verwey, 2009; Ganor-Stern et al., 2013; Kornysheva et al., 2013; Stöcker & Hoffmann, 2004; Verwey, 1999, 2001; 2003b-Experiment 2; Verwey et al., 2016, 2002, 2022a; Wiestler & Diedrichsen, 2013; Yokoi et al., 2017, 2018; Yokoi & Diedrichsen, 2019). Conversely, verbal sequence representations may benefit from DSP practice as shown by a reduced utterance time after having practiced DSP sequences that were initially based on letter series (Verwey et al., 2002). Still, applying well-learned verbal representations is inefficient because they convey little concrete response features (Koch & Hoffmann, 2000b) and the additional processing that is therefore required reduces their contribution to rapidly executed sequences (Cleeremans & Sarrazin, 2007; Ruitenberg et al., 2012a, b; Verwey, 2010, 2015; Verwey & Abrahamse, 2012; Verwey et al., 2009, 2010, 2011; Verwey & Wright, 2014). The notion that verbal sequence knowledge contributes primarily at the start of practice is consistent with participants acknowledging that in the course of practice they had stopped using their prelearned verbal knowledge (Verwey et al., 2002). Quickly abandoning verbal sequence representations is most likely also the reason that this knowledge is easily forgotten (Verwey et al., 2022a). In short, verbal knowledge is commonly not used for executing DSP sequences but it may be applied at the initial practice stages, for example, to prepare the first few responses and the last response.

#### Boosting central-symbolic representations

The studies reviewed below show that sequence learning benefits from practicing without guidance by key-specific stimuli, from repeatedly preparing and imagining the task during mental practice, and from using random practice schedules. As these training procedures all involve STM-based processing and emerge especially with less than about a hundred practice trials their benefits can be ascribed to improved development of central-symbolic representations. Remarkably, these central-symbolic representations appear to result from repeated preparation in STM and physical execution seems not required. Their benefits may increase after practice when the newly developed representations consolidate.

As argued above, in DSP tasks participants usually continue to rely on key-specific stimuli. However, sequence learning appears to benefit when stimuli past the first are either not at all displayed (e.g., De Kleine & Verwey, 2009; Verwey et al., 2002, 2022a) or displayed only when no immediate response is given (Verwey, 2021). Also, sequence learning becomes more independent of key-specific stimuli when practicing with key-specific stimuli of which the second and later ones have the same luminance as the background so that they can be easily ignored (Riesenbeck, 2021).

Indications that central-symbolic representations result from repeated preparation in STM rather than from physical execution come from various DSP studies. This is directly shown by go/no-go DSP studies in which sequences were learned even though they were prepared for only 20 or 40 trials without subsequent execution (Sobierajewicz et al., 2016, 2017a, b, 2018). Also, sequential motor skills developed when participants repeatedly imagined executing motor sequences. Such benefits of *mental practice* have been observed in many real-world motor skills with, for example, athletes and musicians (Allami et al., 2008; Debarnot et al., 2015; Doussoulin & Rehbein, 2011; Gentili et al., 2010; Kraeutner et al., 2017; Zabicki et al., 2016). Mental practice has indeed been argued to promote the development of a ‘perceptual-cognitive action representation’ (Dahm et al., 2023; Frank & Schack, 2017; Ingram et al., 2019; Pascual-Leone et al., 1995), that in the case of physical practice is complemented by a motor component (Allami et al., 2008; Gentili et al., 2006, 2010).

Imagining the execution of DSP sequences arguably involves the same processes as preparing that sequence. Participants who had been instructed to imagine executing the sequence during practice in the case of a no-go signal showed similar learning after 288 practice trials as participants who had been told to just withhold their responses after having prepared it (Sobierajewicz et al., 2016). A strong occipital EEG activity in imagine and preparation groups supported the use of a visuospatial component (reported also by De Kleine & Van der Lubbe, 2011; Van der Lubbe et al., 2021). Still, findings in other studies that mental practice was still more beneficial for learning than sequence preparation (Sobierajewicz et al., 2018; Van der Lubbe et al., 2021) suggest that mental practice prompts more learning than just preparing sequences.

In the contextual interference paradigm (Lee & Magill, 1983, 1985; Shea & Morgan, 1979; Wright et al., 2016) preparation is not explicitly manipulated. Nevertheless, the well-known forgetting-reconstruction hypothesis states that the learning benefit of Random Practice (RP) over Blocked Practice (BP) is due to the repeated need to ‘construct’ a motor plan (also see Lee & Magill, 1983, 1985; Schmidt & Bjork, 1992). It is therefore plausible that the RP benefit is indeed due to preparation in STM with each practice trial that differs from its predecessor. This contrasts with the BP condition in which—like with simple-RT tasks—the sequence can be prepared in STM only at the start of the trial block because information remains available (like with the parameter remapping effect, Rosenbaum et al., 1986, 2007). Indeed, participants took more time during RP than during BP to ‘study’ the imperative stimuli in advance of sequence initiation (Wright et al., 2004). Also, the time to prepare individual sequences reduced more with practice during BP than during RP (Immink & Wright, 1998), and attention demands were lower during BP than RP (e.g., Kim et al., 2018, 2020; Li & Wright, 2000). In line with learning motor sequences by repeated preparation in STM, the typical learning benefit of RP over BP was observed with 4-key DSP sequences after 24 practice trials and not with 7-key sequences that even during BP required successive preparation in STM of the sequence segments (Verwey et al., 2022b). The retention benefit of RP occurred also when 5-element DSP sequences were prepared and not executed in go/no-go DSP studies (Sheahan et al., 2016; Sobierajewicz et al., 2017a, b). Indeed, the BP learning disadvantage disappeared when participants were instructed to evaluate differences with other 4-element sequences before each practice trial (Wright, 1991). Lastly, correlational analyses showed that participants who showed more obvious segmentation in a cycling 9-element DSP sequence—suggesting repeated preparation in STM—also executed these segments more rapidly after about 800 practice trials than those who showed little segmentation and seemed to have relied more on reacting to key-specific stimuli (Verwey, 1996; Verwey & Dronkert, 1996).

Research with the DSP task (Verwey et al., 2022a, b) confirmed indications from other tasks that immediately after practice, the RP learning benefit is limited, and the benefit due to repeated preparation in STM develops mostly after practice has ended (Cohen et al., 2009; Cross et al., 2007; Immink & Wright, 1998; Kim et al., 2016, 2018, 2020; Kuriyama et al., 2004; Lin et al., 2011). Such *off-line consolidation* seems a general phenomenon that also improves reaction skill in a random sequence (Verwey et al., 2022b) and the general skill to produce unfamiliar sequences (Kim et al., 2018; Verwey et al., 2022b). This confirms that an evaluation of different practice strategies should test skill level hours and days after practice has ended.

In conclusion, the results in this section indicate that segments are represented by abstract, length-independent, central-symbolic representations that are most likely coded spatially and sometimes even verbally. These central-symbolic representations result from the repeated preparation of up to about 4 responses in STM that occurs during physical and mental practice. The possibility to prepare the first response in motoric detail in the motor system too and immediately execute it, is held responsible for the reduction with practice of the sequence length effect. With practice this abstract central-symbolic segment representation can be selected by the CP while the preceding segment is being carried out by the MP. Such concurrent selection slows execution of that earlier segment as the CP is then no longer available for the race with the MP to trigger the individual responses. The last sequence element is relatively fast at moderate skill levels because the CP probably uses explicit sequence knowledge to prepare it while the MP is executing the earlier responses of the last segment. The transition between successive segments initially carried out by the CP may gradually become automated when segment representations and their responses become associated too.

### Associative learning

There is a variety of indications that, in addition to the rapid development of central-symbolic representations in STM, sequence execution benefits from associations between representations that are used successively at each of the processing levels. After many hundreds of practice trials, such associative learning starts overshadowing the use of central-symbolic representations. As argued below, indications for the development of associative sequence representations in the DSP task are the reduced reliance on key-specific stimuli towards the end of sequences, the reducing correlations with practice between performance and STM capacity, and the learning of base sequences when participants previously practiced only randomly deviating versions of those sequences. Associative sequence learning at the motor level is demonstrated by the effector-specific component of sequence learning, but associative sequence learning occurs at all processing levels, perhaps even at the level of the retina. The notion that associative learning reduces as there is less temporal overlap in activation explains that in prestructured DSP sequences—with the long RSI separating segments—associative learning between successive segments develops relatively slowly.

As associations are assumed to develop between all representations that are simultaneously active at the perceptual, central (in STM) and motor levels, irrelevant but consistently attended context stimuli also become integrated in the developing sequence representations. This may occur already after tens of practice trials. Display of irrelevant context stimuli seems not to influence sequence performance much unless they have previously occurred with a competing DSP sequence (Shea & Wright, 1995; Wright & Shea, 1991). Notice that the notion that task and sequence execution critically depend on a broad variety of task- and element-specific representations distinguishes associative sequence learning from the classic reflex chaining models that proposed that in behavioral sequences each of a series of movements is triggered solely by the sensory results of the prior movement (Ebbinghaus, 1885; Washburn, 1916, and countered by Henson et al., 1996; Lashley, 1951).

#### Indications for associative sequence learning

A first finding that supports the development of associative sequence learning in DSP sequences is that learning sequences of 3 and 6 responses occurred in elderly and children, even though the 6-key sequences did not show the segmentation that suggests development of central-symbolic representations (Ruitenberg et al., 2013, 2014, 2015; Verwey, 2010; Verwey et al., 2011). Also, the finding that, in a test block, a secondary task tone decelerated slow responses more than fast responses (Verwey et al., 2010) suggests that the faster responses relied more on associative learning than slower responses. Still, in DSP sequences associative sequence learning seems to become dominant only after hundreds of practice trials. This is suggested by segmentation reducing only in the course of 500 and 189 practice trials (Verwey et al., 2022b; Wymbs et al., 2012, respectively). And participants showing no signs of segmentation in a 9-key sequence showed less improvement after 840 practice trials than those who did segment, but disappearance of this performance difference after 1,470 practice trials suggests the eventual development of associative sequence learning (Verwey, 1996). Similarly, the benefit of random over blocked practice after 24 practice trials in contextual interference studies—assumed to be due to improved development of central-symbolic representations—had vanished after 504 practice trials (Verwey et al., 2022b).

Another indicator for gradual associative sequence learning in DSP sequences is that later responses are performed faster and with less reliance on key-specific stimuli than earlier ones. This is in line with activation accumulating across successive sequence elements (MacKay, 1982). Retrospective inspection of RT curves suggests such a gradual speed increase over successive responses in unstructured 6- and 7-key DSP sequences after about 500 practice trials (Ruitenberg et al., 2015; Verwey, 2021; Verwey et al., 2009, 2010; Verwey & Wright, 2014) while this was not observed after about 200 practice trials (Barnhoorn et al., 2019a, b; De Kleine & Verwey, 2009; Ruitenberg et al., 2015; Verwey, 2010).^5^ As these observations were not statistically tested, a more convincing finding is that after hundreds of practice trials reliance on key-specific stimuli significantly reduced towards the end of DSP sequences when key-specific stimuli were not displayed at all (Verwey, 1999; Verwey et al., 2022b), were displayed only occasionally (Verwey, 2021), and when they consisted of neutral *X*s (Verwey et al., 2020). A reducing reliance on later key-specific stimuli was observed also when sequences involved an incompatible S–R mapping (Riesenbeck, 2021), when a secondary task was carried out (Brown & Carr, 1989), when preparation was reduced by executing an immediately preceding sequence (Verwey, 1999), and by expecting deviating stimuli (Verwey & Abrahamse, 2012; Verwey & Wright, 2014; see similar findings in Ganor-Stern et al., 2013; Ruitenberg et al., 2014). Furthermore, the correlations between learning rate and segmentation pattern with visuospatial STM capacity disappeared with practice (Seidler et al., 2012). Evidence that associations prime more than just the ensuing response is that DSP sequences were still executed faster than unfamiliar sequences when after practice only keys at odd sequence positions had been changed (Verwey, 2003b), and when deviating stimuli occurred at random locations during practice (Verwey & Wright, 2014, like in the alternating SRT task, e.g., Howard & Howard, 2001). Convincing evidence for associative sequence learning was that practicing sequences that always included a deviating element from the underlying, base, sequences still led to learning that underlying sequence (Verwey & Wright, 2014).

Another indication for associative sequence learning in DSP sequences is that, like in the SRT task (Frensch & Miner, 1994; Soetens et al., 2004), after substantial practice skill in the DSP task benefits from high execution rates (Barnhoorn et al., 2019a, b; Verwey & Dronkers, 2019; also see Deffains et al., 2011; Stadler, 1995). This particular effect is consistent with successively used response representations becoming associated when they are repeatedly coactive before activation decays (Cowan, 2005; Frensch & Miner, 1994; McLean & Shulman, 1978; Mueller et al., 2003). That this does not occur with central-symbolic representations in earlier practice can be attributed to those associations between responses developing in the preparation and not in the execution phase (Mantziara et al., 2021; Verwey & Dronkers, 2019). Reduced learning with longer intervals, in early and after substantial practice, also explains the limited transfer of sequence training when the temporal structure is changed (e.g., from ABC-DEF to BCD and CDE; Verwey, 1996; cf. Stadler, 1993; Verwey & Dronkert, 1996).

In line with perceptual sequence learning in SRT tasks (Abrahamse et al., 2010; Song et al., 2008; Toh et al., 2022; but see, Deroost & Coomans, 2018), two pilot DSP studies suggest that associative sequence learning may even occur in terms of retinal locations. In these studies, changing the fixation location on the display from the right to the left of the stimulus area implied a relocation of the three stimulus placeholders from the left to the right peripheral visual field (Giusti, 2021; Rödig, 2009). Participants were required to detect color changes of the fixation cross to prevent eye movements away from the fixation cross. This fixation relocation resulted in slowed execution of familiar sequences after 450 practice trials, but not after 50 practice trials and with unfamiliar sequences. So, stimuli seem to be identified more quickly when they are in the same general retinal location as during practice. This can be explained by the learning of retinotopic maps throughout the visual system (see review by Groen et al., 2022) and by stimulus location order learning at the retinal location used (cf. Shiu & Pashler, 1992).

In the SRT task, associative sequence learning at the motor level was further indicated by an effector-dependent component after hundreds of practice trials (Berner & Hoffmann, 2009a, b; Deroost et al., 2005; Verwey & Clegg, 2005). Similarly, in DSP sequences effector-specific sequence learning was observed when other fingers were used after about 500 practice trials (Verwey et al., 2009; Verwey & Wright, 2004). And limited transfer of sequence learning when very different response modalities were used suggested effector-specific sequence learning already after 148 practice trials (Barnhoorn et al., 2016). A neurophysiological indication that visuospatial coding is used less in the course of practice is the stronger CDA component in the EEG during preparation and execution of unfamiliar 5-key sequences as compared with familiar 5-key sequences (Sobierajewicz et al., 2016). An important indication that effector-specific sequence learning is indeed based on associative sequence learning at the motor level is the increasing disadvantage of using other fingers at later sequence positions in familiar sequences (Verwey et al., 2009). A possible indication that associative sequence learning may occur also at the level of hand-independent finger identities (e.g., between ring and index fingers), is that sequencing skill has repeatedly been found to transfer to a mirror sequence performed with the other hand after one-handed practice (Bapi et al., 2000; Deroost et al., 2005; Grafton et al., 2002; Gruetzmacher et al., 2011; Panzer et al., 2009; Verwey & Clegg, 2005; Wiestler & Diedrichsen, 2013; Wiestler et al., 2014). This transfer to mirror sequences is not observed after two-handed practice. Yet an alternative account is that motor sequence learning involves series of hand postures (Graziano, 2006; Verwey, in press-a) that can also be used by the other hand.

Finally, the development of associations may be responsible also for the automated concatenation of successively used central-symbolic representations in unstructured sequences with their relatively short inter-segment intervals. This explains the gradual fastening of the slow concatenation responses with extensive practice (Kennerley et al., 2004; Popp et al., 2020; Ramkumar et al., 2016; Verstynen et al., 2012; Verwey et al., 2022b). Such association development may also explain the increase in average segment length across participants groups after hundreds or thousands of practice trials (Acuna et al., 2014; Greeley et al., 2020, 2022; Ruitenberg et al., 2012a, b; Wymbs et al., 2012).

#### Context effects

It has long been known that memory retrieval is better in the situation in which learning originally took place. This *context effect* is attributed to knowledge in memory becoming associated with representations of context stimuli that are irrelevant to the task itself (Smith, 1988). This relates to findings of cells in prefrontal cortex showing sensitivity to a conjunction of a stimulus and its context (Dang et al., 2021). With respect to DSP sequences, a distinction can be made between dynamic and static context stimuli.

Dynamic context stimuli vary with each sequence element and may consist of (external) response feedback and the representations of preceding sequence elements. With practice, they are likely to become incorporated in the sequence representations by mere temporal contiguity (Frings et al., 2007). Inspired by two earlier DSP studies (Shea & Wright, 1995; Wright & Shea, 1991), a typical DSP study was carried out that involved about 500 trials per sequence and then manipulated the irrelevant color, shape, and general screen location features of key-specific stimuli (Ruitenberg, Verwey & Abrahamse, unpublished work). In contrast to the Wright and Shea studies, changing the irrelevant context features after practice did not affect sequencing performance. In retrospect, the substantial amount of practice and the permanent availability of the stimuli during execution may have allowed the participants in the latter study to learn to ignore irrelevant stimulus features so that they were not incorporated in the sequence representations (Verwey et al., 2020). Future research should explore whether context effects may indeed develop with limited practice and can be overcome with more extended practice by an increased focus on just the relevant stimuli.

Static context stimuli are irrelevant stimuli that do not change during sequence execution but still affect performance when changed, such as the working environment (Godden & Baddeley, 1975), background music (Smith, 1985), and features of the imperative stimuli that are displayed before sequence execution (Wright & Shea, 1991). Changing the background photo with which a DSP task was practiced for 500 practice trials failed to show a context effect (Ruitenberg, Abrahamse & Verwey, unpublished work), suggesting again that during practice participants learned to ignore the irrelevant background stimulus. Still, execution of 3- and 4-key DSP sequences in other studies was slowed when after 36 practice trials static, irrelevant features of the imperative stimuli were displayed that had previously been used with a competing sequence (Shea & Wright, 1995; Wright & Shea, 1991). These features involved the color, shape and screen location of the placeholders that were all displayed simultaneously before sequence initiation. These context changes appeared to slow initiation of the 3-key, and execution time of the 3- and 4-key sequences after practice with shorter (300 to 600 ms) preparation times (Anderson et al., 1998). No such context effects were found with longer (400 to 800 ms) preparation times. These two studies suggest that after limited practice focusing on relevant stimulus features involves time-consuming suppression of irrelevant stimulus features. The finding that this effect of static context stimuli on sequence execution was larger for 4- than 3-key sequences supports that limited preparation time forces participants to retrieve responses using context-dependent memory retrieval only after they have initiated the first response (Wright & Shea, 1991). A study with the dit-dah paradigm (see “Keying sequences” section) confirmed that after only 24 practice trials preparing each response of 4-element timed keying sequences (also called the SEQ process; Klapp, 1995, 2003) involved context-dependent LTM retrieval. Participants seem to have used the longer intervals to retrieve representations from LTM. Instead, response duration planning (i.e., the INT process) was not context dependent.

Two go/no-go DSP studies involved executing two 6-key sequences after all six key-specific stimuli had successively been displayed for 750 ms each. Ruitenberg et al. (2012a, b) showed with this paradigm that after 50 and 250 practice trials reversing the irrelevant color of these stimuli slowed both concatenation and execution responses. A similar go/no-go DSP study showed the context effect after 300 but not after 50 practice trials (Ruitenberg et al., 2015). Given the obvious need in this task to prepare these DSP sequences in STM, these results confirm that irrelevant stimulus features can with tens or hundreds of practice trials become integrated into central-symbolic sequence representations. The context effect may have occurred here because the 750 ms display time was insufficient to suppress irrelevant features of the key-specific stimulus.

So, the above studies show that, in addition to the development and use of central-symbolic representations, the skilled execution of DSP sequences gradually also benefits from associative learning at each processing level. These associations are dependent on a context consisting of prior sequence elements, the sensory feedback they induce, and active task- and goal-specific representations. The resulting activation accumulates and benefits later responses in discrete motor sequences more than earlier ones. Associative learning at the motor level is responsible for the effector-specific component of sequence learning.

### Explicit sequence knowledge

The interest in participants’ awareness of their motor sequences is fueled by the repeated observation that explicit sequence knowledge improves sequence execution. The DSP studies below show that this benefit occurs primarily in sequences executed at moderate execution rates, most likely because these sequences provide the time to develop and apply explicit knowledge. Explicit knowledge of the earlier responses is used for preparing responses in STM in early practice which influences the development of the segments. The assessment of explicit sequence knowledge appears heavily influenced by the participants’ capacity to reconstruct their sequences. Some participants are better able and/or more motivated to reconstruct their sequences than others and this is reflected also in their performance of novel and random sequences. In DSP tasks, explicit knowledge seems to be primarily of a spatial nature, even when practiced sequences originally involved verbal sequence knowledge. Explicit sequence knowledge basically develops unintentionally when practicing DSP sequences and involves especially the first and last responses, but some participants appear to intentionally improve their explicit sequence knowledge by actively developing and testing hypotheses about the order of the sequence elements. Still, explicit sequence knowledge can also be quickly forgotten when no longer used.

#### Assessing explicit sequence knowledge

Across five DSP studies only 47% of the participants was able to correctly write down both their sequences (Verwey et al., 2016). In addition, 68% of the participants in those studies indicated that they had reconstructed their sequences during the test by tapping them on the table or in their mind (like when imagining to execute the sequences, Sobierajewicz, et al., 2017a, b). The latter shows that writing down sequences in awareness tests does not show the amount of immediately available explicit sequence knowledge, as performance on this awareness assessment task is boosted by participants taking the time to reconstruct sequence order using episodic memories and implicit sequence knowledge.

A more recently developed computerized awareness assessment task was used that required participants to successively click with a mouse the locations or letters of the keys they had been pressing to execute the keying sequences (Verwey & Dronkers, 2019; Verwey et al., 2020, 2022a, b). It showed lower awareness than the above paper-and-pencil test in that only 17% of the participants in Verwey and Dronkers (2019), 19% in Verwey et al. (2022a), and 31% in Verwey et al. (2022b) showed full awareness of the response locations used in both sequences. This shows that the type of awareness assessment task affects sequence reconstruction. Participants in these studies also indicated to have played off during the awareness test their implicit sequence knowledge to determine the response order (Verwey et al., 2022a).

The computerized awareness test further indicated that in DSP sequences explicit knowledge relies more on visuospatial than on verbal coding (Verwey & Dronkers, 2019; Verwey et al., 2022a, b). The notion that participants tended to code explicit sequence knowledge in terms of successive locations is consistent with the finding that they seemed to still develop and use spatial explicit sequence knowledge when sequence execution was based on responding to letters instead of spatial stimuli (Verwey et al., 2020), and when they initially used learned letter series instead of visual stimuli (Verwey et al., 2022a).

#### Developing explicit sequence knowledge

Explicit sequence knowledge is likely to benefit from repetition by covert rehearsal in STM and repeated knowledge application. This would explain that awareness was higher after a Random Practice (RP) than a Blocked Practice (BP) regime in contextual interference studies with 24 practice trials (Verwey et al., 2022b), that participants were better able to utter previously learned letter series after, than before, 210 practice trials (Verwey et al., 2002), and that awareness was greater for participants practicing for 504 than 24 practice trials (Verwey et al., 2022b). The notion that the development of explicit sequence knowledge demands CP and STM capacity can account for participants sometimes possessing full explicit knowledge of one and not of the other practiced DSP sequence (Verwey, 2015; for similar findings in the SRT task see Esser et al., 2022; Haider et al., 2011).

A number of DSP studies involved participants intentionally learning explicit sequence knowledge in terms of letters or number series and then using that knowledge in response to display of a sequence-specific stimulus (Brown & Carr, 1989; De Kleine & Verwey, 2009; Verwey, 1999, 2001; Verwey et al., 2002, 2016, 2022a; Yokoi & Diedrichsen, 2019). Like episodic memories, this type of explicit knowledge appears volatile in that awareness had reduced already after 1 day (Verwey et al., 2022a). Most likely, participants stop using explicit sequence knowledge when more suitable sequence knowledge develops (Verwey et al., 2002). Displaying key-specific stimuli first in go/no-go DSP tasks, either successively (Ruitenberg et al., 2012a, b; Ruitenberg et al., 2015) or simultaneously (Shea & Wright, 1995; Wright & Shea, 1991), would seem to benefit explicit sequence knowledge but that still did not always provide full awareness (Ariani & Diedrichsen, 2019). A good way to stimulate development of explicit sequence knowledge in DSP sequences appears to involve displaying key-specific stimuli only when after 800 ms a response has not yet been given (Verwey, 2021). This probably helps participants to develop and test their hypotheses on response order (Esser et al., 2022; Haider & Frensch, 2009).

The amount of explicit sequence knowledge differs greatly amongst participants, and in most DSP studies only a minority of participants shows full explicit sequence knowledge. The relatively good knowledge of the first few and last sequence elements (Verwey, 2015; Verwey & Wright, 2014) can be considered primacy and recency effects like in episodic and semantic memory (e.g., Bonanni et al., 2007; Johnson, 1991). The reason that participants with a larger STM capacity remember more sequence elements (Barnhoorn et al., 2016; Bo et al., 2009; Bo & Seidler, 2009; Seidler et al., 2012) may well be that they (are better able to) use the strategy to intentionally develop and test hypotheses about response order (Cleeremans & Sarrazin, 2007; Esser et al., 2022; Frensch & Rünger, 2003; Haider & Frensch, 2009; Lustig & Haider, 2019).

#### Applying explicit sequence knowledge

Sequence performance and performance on awareness tasks showed significant correlations after 200 but not after 500 practice trials (Ruitenberg et al., 2012a, b; Verwey, 2010, 2015; Verwey & Abrahamse, 2012; Verwey et al., 2009, 2010, 2011; Verwey & Wright, 2014; cf. Cleeremans & Sarrazin, 2007). These correlations are significant also when sequences are executed more slowly for other reasons than limited practice, like when participants expect a deviating stimulus in familiar sequences (Verwey, 2015; Verwey & Abrahamse, 2012), and when sequences are not guided by key-specific stimuli past the first (Verwey, 2010; Verwey et al., 2022b). These findings indicate that explicit sequence knowledge contributes to executing DSP sequences, especially when there is sufficient time to utilize that knowledge for selecting and triggering individual responses (Cleeremans & Sarrazin, 2007). The application of explicit knowledge to improve sequence execution seems strategic in that participants indicated that they stopped using explicit knowledge after a while when sequences were executed more rapidly (Verwey et al., 2002). Also, sequence execution benefits of explicit knowledge have been observed only when participants had explicit knowledge of both sequences, and not of only one (Verwey et al., 2010), as if participants refrained from preparing to apply explicit sequence knowledge when they did not have full explicit knowledge of both sequences (cf. Esser et al., 2022).

When participants have only partial awareness this usually concerns the first few and the last responses (Verwey, 2015; Verwey & Wright, 2014). This knowledge is probably responsible for the faster first few responses and last response in more aware participants after 90 practice trials (and indeed not after 540 practice trials; Verwey & Wright, 2014) and in another study across practice trials 360 to 540 (Verwey & Abrahamse, 2012). Participants probably use this explicit knowledge to prepare the first responses before sequence initiation and the last response while executing the preceding responses. The role of explicit sequence knowledge in preparing a few responses before executing them is consistent with more aware participants also showing more enhanced segmentation (Verwey, 2015; Verwey & Abrahamse, 2012; Verwey et al., 2010; and in preadolescent children: Ruitenberg et al., 2013).

An important last observation was that participants with more awareness were also faster producing novel and random sequences (Verwey et al., 2022b). Perhaps, more aware participants may have superior processing capabilities, like a larger STM capacity (Seidler et al., 2012) and a higher processing speed (Salthouse et al., 1998), and also more motivation. Indeed, individuals with a higher STM capacity developed longer segments and executed DSP sequences at higher execution rates (Barnhoorn et al., 2016; Bo et al., 2009; Bo & Seidler, 2009; Seidler et al., 2012). Conversely, older participants with their lower processing rates were slower executing sequences and processing information, and were therefore probably less able to develop and apply explicit sequence knowledge (e.g., Barnhoorn et al., 2016; Verwey, 2010; Verwey et al., 2011).

Taken all together, one could say that explicit sequence knowledge helps in shaping central-symbolic representations and segmentation of sequences by its initial contribution to sequence preparation in STM. Immediately available explicit knowledge is less than suggested by awareness tests and its contribution to skilled sequence execution is limited by the time it takes to reconstruct and apply that knowledge. Individual awareness differences can be attributed to superior processing capacity and to deliberate activities to develop explicit sequence knowledge.

### General skills

It is well-known that during practice perceptual-motor skills become increasingly task-specific and that this reduces the transfer of practice effects to modified versions of the task (Ackerman, 1988; Blandin et al., 2008; Fleishman & Hempel, 1955; Proteau et al., 1992). This recognized finding fits with indications in DSP studies that practice generates not only sequence-specific, but also more general skills (Verwey, 2010). The studies below confirm for the DSP task the development of the skills (a) to react to key-specific stimuli in DSP sequences; (b) to optimize processing strategies including advanced preparation, concurrent processing of information, and manual dexterity; and (c) to produce DSP sequences with complicated rhythmic patterns.

#### Reaction skill

Several DSP studies show that frequently and consistently reacting to key-specific stimuli improves the contribution to sequence execution of key-specific stimuli in general. This is indicated, for example, by the finding that random sequences were executed faster after 504 practice trials with fixed DSP sequences than after 24 such practice trials (Verwey et al., 2022b). Random sequences were also executed faster after participants most likely had relied on selecting responses on the basis of key-specific stimuli. That is, random sequences were executed faster after practice of DSP sequences had involved increased focusing on key-specific stimuli (1) because deviating stimuli were occasionally displayed (Verwey & Wright, 2014), (2) when participants had been instructed to be extremely accurate (Barnhoorn et al., 2019a, b), and (3) when prior practice had involved several sequences in a block of trials (in Random Practice) as opposed to trial blocks including a single sequence (in Blocked Practice; Verwey et al., 2022b). Finally, elderly appeared to largely transfer the sequencing skill they had developed across 144 practice trials to unfamiliar sequences (Verwey, 2010; Verwey et al., 2011) indicating that their improvement of the practiced sequences was based more on learning to react than on learning response order in these sequences. These studies do not make clear whether this reaction skill is specific for the stimuli and responses of the DSP sequences performed or extends also to other stimuli or responses. The finding that the experienced pianists amongst the participants in one DSP study executed unfamiliar sequences faster than the nonmusicians (Verwey, 2015) suggests that reaction skill need not be restricted to familiar DSP stimuli and responses. However, a recent study suggests that with extended practice, participants also learn to respond specifically to the feature of a stimulus that is available the earliest (Verwey, in press-b).

In addition to selecting individual responses, participants also appear to develop the skill to select entire sequences. This is demonstrated by the slowed initiation of 2- and 6-key sequences when after 500 practice trials the mapping between sequence-specific stimuli and the practiced sequences was reversed, even after blocked practice (Verwey, 1999). This corroborates the development of integrated sequence representations which, irrespective of their length, can be selected after practice, just like individual responses.

#### General sequencing skill in DSP tasks

The index for general sequencing skill is improved execution of unfamiliar sequences while this cannot be attributed to improved reaction skill. For example, novel 4-key sequences were performed better than random sequences when other sequences had first been practiced for 24 trials, and novel 4-key sequences were executed faster in a blocked condition after practicing for 504 trials DSP sequences in a BP than in a RP regime (Verwey et al., 2022b). This cannot be attributed to improved reaction skill as the unfamiliar sequences would then have been executed faster after RP than BP. Also, practicing four 5-key sequences for 480 trials each in a go/no-go study benefited unfamiliar sequences relative to a pre-practice phase (Wiestler & Diedrichsen, 2013). This cannot be attributed to a reaction skill either as the go/no-go paradigm does not involve immediate reactions. Novel 6-key sequences were executed faster after about 500 practice trials with normal DSP sequences than when during practice sequences had involved randomly occurring deviating stimuli (Verwey & Wright, 2014). And transfer of prior practice with DSP sequences to novel DSP sequences was higher after practice with 0 RSIs than with RSIs of 500 ms or more (Verwey & Dronkers, 2019; cf. Deffains et al., 2011). These indications for improved execution of unfamiliar sequences after practice of other DSP sequences cannot be attributed to reaction skill. This suggests that general sequencing skill includes the ability to employ sequence knowledge in a particular situation (like in BP or RP), by for example improving preparation and the timing of the processes used.

Execution of DSP sequences also seems to involve skills that exceed experience with DSP sequences in that skilled video gamers initiated DSP keying sequences faster at the start of practice while execution rate was faster in all practice blocks (Verwey & Wright, 2014; cf. Romano Bergstrom et al., 2011; Rosenthal et al., 2011). And participants who played, or had played, piano were faster producing unfamiliar DSP sequences (Verwey, 2015). Similarly, professional pianists executed unfamiliar and familiar 5-element DSP sequences in a go/no-go DSP task faster and more accurately than nonpianists (Sobierajewicz et al., 2018). This was attributed to better manual dexterity of the pianists because the benefit occurred during execution and the nonpianists eventually made up by improving more than the pianists. That group differences need not only be caused by processing differences was shown by the finding that the slower responses of older participants were caused in part by one or more structurally slow fingers (Barnhoorn et al., 2019a, b).

#### Execution rate and timing

In line with the tradition to trace cognitive processes by assessing RTs under speed instructions (Sanders, 1998; Sternberg, 1969), DSP sequences are typically executed at maximum execution rates while keeping error rates reasonably low. Motor sequence research has, however, also studied sequence execution at low rates assuming that execution rate can be specified by setting a speed parameter (Schmidt, 1975; Shea & Wulf, 2005). In associative sequence models execution rate can be specified by manipulating the threshold at which representations fire (Bogacz et al., 2010; Brown & Heathcote, 2008). The chosen execution rate usually seems specified on the basis of prior experiences and accuracy demands, but it may be strategically adjusted too (Wong et al., 2017; also see Riesenbeck, 2021; Thura & Cisek, 2017). Importantly, the assumption that different representations underlie sequence skill implies that changing the set execution rate also changes the relative contributions to motor execution of processes and representations. As argued earlier, at lower rates there is a larger contribution and faster development of explicit sequence knowledge. At higher rates there is a larger contribution and faster development of associative, implicit sequence knowledge (Barnhoorn et al., 2019a, b; Verwey & Dronkers, 2019).

With respect to the differential timing of individual sequence elements, a distinction can be made between the spontaneous development of a specific timing pattern due to segmentation and learning a specific rhythm. In rapidly executed DSP sequences, the spontaneously developing temporal pattern is sequence-specific and reflects the succession of segment representations (Verwey et al., 2009, 2010; Verwey & Eikelboom, 2003). Conversely, imposing a rhythm onto a fixed sequence by introducing a pause may influence segmentation (Verwey, 1996; Verwey & Dronkert, 1996; Verwey et al., 2019). In rhythmic sequences, execution rate is usually submaximal, and separate processes control response order and temporal structure (Bengtsson et al., 2005; Kornysheva et al., 2019; Mantziara et al., 2021; Shin & Ivry, 2003; Ullén & Bengtsson, 2003). In these sequences, response order and timing can be prepared at different moments (Klapp, 2003; Maslovat et al., 2016). This independency makes memory storage more efficient (Kornysheva & Diedrichsen, 2014; Schmidt, 1975) and allows independent transfer of ordinal and temporal sequence properties to unfamiliar sequences (Bortoletto et al., 2011; Kornysheva & Diedrichsen, 2014; Kornysheva et al., 2013; Maslovat et al., 2018; Ullén & Bengtsson, 2003). Still, in line with context-dependent memory retrieval, timing appears closely associated with a particular sequence (Verwey et al., 2009), and with a specific action systems (Buhusi & Meck, 2005; Ivry, 1996; Shin & Ivry, 2002; Wong et al., 2017). Different systems seem responsible for timing intervals of tens to hundreds of milliseconds and for timescales of seconds (Mauk & Buonomano, 2004). When the rhythm involves several different intervals these intervals can probably not be prepared concurrently and they need to be specified separately during sequence execution (Kleinman et al., 2016; Maslovat et al., 2018).

So, it appears that practicing DSP tasks yields several general skills that benefit other DSP sequences too. These include the skills to improve reacting to key-specific stimuli, optimizing advanced preparation, using concurrent information processing, improving manual dexterity, and applying complicated rhythmic patterns.

### Executive control

RT research often does not take into account the importance of task preparation. However, the processing system needs to prepare central and motor processes to achieve the virtually automatic translation of a stimulus into a response (cf. procedural working memory suggested by Monsell & Driver, 2000; also see Hommel, 2000). This preparation involves, first of all, activating task and goal representations by the CP to set the context that is essential for retrieving the required information and processes from LTM (Logan & Gordon, 2001; Memelink & Hommel, 2013; Miller & Cohen, 2001; Rangelov et al., 2013). Indeed, prefrontal neurons have been found to encode task-dependent context stimuli too (Dang et al., 2021). In the DSP task, the importance of general preparatory processes is indicated by the finding that the sequence length effect suddenly appeared in choice tasks too when preparation was hampered by a preceding choice-RT task (Verwey & Eikelboom, 2003) or by a preceding sequence (Verwey, 1996, 1999). The sequence length effect even occurred in a few choice-RT studies when preparation was not hampered (Brown & Carr, 1989; Schröter & Leuthold, 2008; Stöcker & Hoffmann, 2004; Verwey, 1994a, 2010; Verwey & Eikelboom, 2003).^6^ suggesting individual and task-specific differences in preparation. Also, DSP sequences were initiated more slowly when after normal practice sequences were to be executed in the reaction mode, as if sequencing processes were automatically prepared and then had to be suppressed (Verwey & Abrahamse, 2012).

It lies at hand that the required processes and their order can be prepared and held active for later use, just like the individual responses of a DSP sequence. This is suggested by two DSP studies in which high- and low-pitched tones were displayed during execution of a DSP sequence and participants were to count the low-pitched tones (Verwey et al., 2010, 2014). Slowing of the three or four responses following each tone indicated that tone display made the CP temporarily switch from triggering responses to tone processes that had been prepared while the MP and the associative sequencing mechanisms continued executing the sequence. When instead tones were to be ignored responses of the sequence were not slowed showing that in that situation tone processes had not been prepared. Detailed examination of response slowing showed that the tone identification process was set to be immediately followed by incrementing the tone counter when tones were easy to identify (Verwey et al., 2014) while counting probably occurred only after sequence completion when the tones were harder to identify (Verwey et al., 2010). Hence, task differences determined the order and timing of the CP executing the required processes.^7^

Two DSP studies suggest that the order of processing stages can automate within less than 100 trials. When instructed to ignore tones sequence initiation slowed more as participants had more experience with tone counting as if with practice the tone counting processes were automatically invoked and needed increasing suppression (Verwey et al., 2014). Similarly, familiar DSP sequences were initiated more slowly when after normal DSP practice participants were expecting deviating stimuli (Verwey & Abrahamse, 2012). They apparently had to suppress the automatic tendency to produce familiar DSP sequences in a sequencing mode and rely again on the reaction mode.

In the past, researchers have argued that processing is automatic when it occurs without intention, the performer is not aware of these processes, and performance is not affected by a secondary task (Ashby & Crossley, 2012; Logan, 2018; Moors & De Houwer, 2006; Saling & Phillips, 2007). According to these three criteria skilled execution of DSP sequences cannot be considered automatic, just like manual gear shifting never seems to become entirely automatic (Shinar et al., 1998). After all, the reviewed DSP studies indicate that awareness of the sequences is highly different across individuals, and that a tone counting task always slowed the four responses that followed each tone (Verwey et al., 2010; Verwey et al., 2014). It is also unlikely that these DSP sequences were executed without the intention to do so. Nevertheless, the capacity of the MP and the associative mechanisms to execute successive responses, once initiated, does meet a fourth criterion for automatic processing. This is that, once initiated, an automatic process runs autonomously and ballistically, without cognitive monitoring (Bargh, 1992; Tzelgov, 1997, 1999). The above findings suggest that the control of processes underlying execution of familiar DSP sequences are automatic according to this criterion. This automatic triggering of inappropriate processes in a task also seems responsible for the occurrence of action slips (Botvinick & Bylsma, 2005; Norman & Shallice, 1986; Reason, 1992).

So, in line with the notion that people can learn fixed response sequences, in a well-known task they also seem to be learning the order of processes. This is reasonable considering that responses in a sequence result from successive processes too. Improved processing sequences is suggested by indications that counting and response triggering processes carried out by the CP could be prepared and retained until needed, and that deviations from the learned processing order by removing or introducing deviating stimuli and removing a secondary task slowed sequence initiation. Skilled sequence execution is not automatic according to the three traditional criteria for automaticity, but it can be considered automatic in that, once initiated, the execution of the processes may proceed autonomously after practice.

## C-SMB 2.0

Practicing DSP sequences involves a gradual transition from responding to one of two short series of key-specific stimuli in the *reaction mode* to a 2-choice RT-task in which each response consists of a familiar keying sequence that is rapidly executed in a seemingly effortless way. The present article is aimed at unveiling the cognitive processes and memory representations that are responsible for this transition. This is important for understanding how sequential motor skills develop and also unveils general principles of the cognitive system. Earlier research with the DSP task led to the Dual Processor Model (Verwey, 2001; further worked out in Abrahamse et al., 2013) and C-SMB (Verwey et al., 2015). On the basis of the phenomena shown by the above review, this section proposes their successor the second version of the Cognitive framework of Sequential Motor Behavior, or C-SMB 2.0. As the amount of practice determines the contribution of the various processing mechanisms and sequence representations the descriptions of the various processes and mechanisms in this section are accompanied by rough indications of the number of practice trials at which those mechanisms and representations come into play.

### Assumptions of C-SMB still standing

In line with the research philosophy that cognitive models need to rely on similar theoretical constructs, the first version of C-SMB was inspired by the processing architectures suggested by the Additive Factors Model for choice-RT tasks (Sanders, 1990, 1998; Sternberg, 1969) and the related bottleneck model for the psychological refractory period task (Pashler, 1994). It was also inspired by the parallel processing architecture of the neural processing system. C-SMB’s core assumptions were the use of separate processors and that learning is associative. These are the core assumptions of C-SMB 2.0 as well.

The processors postulated by C-SMB were the *central processor* (CP) that gets its input from modality-specific *perceptual processors* (PPs) and that transmits its output to modality-specific *motor processors* (MPs) to produce manual or verbal responses. The CP was, and still is, held responsible for performing in choice-RT tasks the central processing stages of ‘stimulus identification’, ‘response selection’, ‘parameter specification’, and ‘motor unpacking’ (Sanders, 1990; Sternberg, 1969; Verwey et al., 2015). The earlier perceptual and later motor processing stages were assumed to be carried out by specialized modality-specific perceptual and motor processors. This architecture of separate processors each executing several processing stages is important as it can account for the many indications that perceptual, central and motor processes can concur (e.g., Pashler, 1994; Sigman & Dehaene, 2005).

The development of associations between representations in LTM being responsible for knowledge storage was considered central to learning. These associations were assumed to develop when representations are repeatedly co-active, much in line with the classic postulate that “neurons wire together if they fire together” (Hebb, 1949; Lowel & Singer, 1992). In C-SMB these associations are responsible for the development of (1) the *S–R associations* that underlie a reaction skill that develops independently of any sequence, (2) *central-symbolic representations* that allow for a cognitive loop that selects successive abstract response representations in STM, and (3) *associative sequence learning* that allows for the successive triggering of concrete response representations in a motor loop. In retrospect, these cognitive and motor loops are much like the outer and inner loops proposed for typing skill by Logan and Crump (2011). The slowing of a segment when it is followed by another segment was attributed to the CP preparing the oncoming segment as that eliminates the contribution of the CP to the race with the MP when triggering the individual responses. Longer sequences were assumed to involve concatenation of both central-symbolic and motor chunk sequence representations.

### Introducing C-SMB 2.0

The above review of DSP studies discusses results that corroborate the above assumptions of C-SMB, but it also suggests several interesting new assumptions that together merit the proposal of C-SMB 2.0. These new assumptions are described in detail below and involve, in short, (1) the lasting contribution of key-specific stimuli, (2) that the development of central-symbolic representations is caused by repeated preparation in STM and that sequence-specific representations and general sequencing skills consolidate after practice, and (3) that segmentation is caused solely by the limited capacity of STM. Also, (4) the development of associative sequence learning occurs at all processing levels and it results from actual sequence execution. Associative learning at the motor level is responsible for the effector-specific component of sequence learning. Other novel assumptions concern (5) the possibility that irrelevant stimuli become part of the context and may later influence use of sequencing skills, and (6) that preparation of DSP sequences occurs not only in STM but that a single response can be prepared in detail at the motor level too. Also, (7) concurrent selection of the next segment and the slowness of the concatenation responses indicate that central-symbolic representations can be selected as a whole but that storing them in STM involves further specification which is basically possible only after STM is no longer occupied for executing the preceding segment. Further assumptions pertain to (8) the way explicit sequence knowledge develops and is utilized, and (9) the notion that practicing DSP sequences also induces general skills that are not specific for the practiced sequences. In contrast to C-SMB, C-SMB 2.0 does not assume that sequence learning at the motor level involves preparation in a limited capacity motor buffer that would eventually produce motor chunks in the sense of concrete response representations. Table 1 provides an overview of the most important properties of C-SMB and C-SMB 2.0.

**Table 1:** Overview of the defining features of C-SMB and C-SMB 2.0, and their differences

	C-SMB	C-SMB 2.0
GENERAL		
C-SMB is evidence-based	√	√√
Indications are given of the amount of practice needed	-	√
SEQUENCE EXECUTION		
The processing architecture consists of perceptual, central, motor processors	√	√
2 Hierarchical control levels: cognitive and motor loops	√	√
Preparation of abstract response representations in STM prompts central-symbolic representations and segmentation	(√)	√
Preparation may involve several STM components (spatial, verbal, episodic)	-	√
Central-symbolic representations are prepared as abstract response representations in STM	(√)	√
Preparation of concrete response representations in a short-term motor buffer prompts motor chunk development	√	-
Continued use of displayed key-specific stimuli via S–R associations (CP_SR_)	-	√
A single response is prepared in detail at the motor level—immediate execution eliminates the sequence length effect	-	√
Detailed processing descriptions of DSP sequence execution in blocked (simple-RT) and random (choice-RT) conditions	-	√
CONCURRENT PROCESSING		
Concurrent processing of CP and MP allows racing processors	√	√
Fast execution of last response due to CP using explicit knowledge	-	√
Slow concatenation response due to preparing a central-symbolic representation (specifically) in STM	-	√
LEARNING		
Central-symbolic representations are multimodal representations like event and task files	(√)	√
Segmentation determined by preparation (of explicit knowledge) in STM	(√)	√
Segmentation determined by preparation in the motor buffer	√	-
Sequencing skill includes general sequencing skills and reaction skill	-	√
Associative learning at the central processing level, independent from STM	-	√
Development of central-symbolic representations results from repeated preparation in STM, which is strengthened by off-line consolidation	-	√
Associative learning allows independence from CP control once initiated, and this increases across successive sequence elements due to accumulating activation	-	√
Associative motor learning responsible for effector-specific sequence learning	-	√
Context-dependence of associations, including those in central-symbolic representations, result from prior elements as well as from attention during practice	-	√
STM load is determined by the number of stored response representations, irrespective of whether they are part of a central-symbolic representation	-	√
Central-symbolic representations can become associated across short intervals—reducing indication for segmentation	-	√
Awareness testing is influenced by a reconstruction using implicit/episodic knowledge	-	√
Awareness develops initially at the sequence start and end	-	√
Awareness test performance is influenced by general processing abilities	-	√
EXECUTIVE CONTROL		
Executive control is based on a context that includes goal representations and prior behavior	-	√
Control of successive processes may automate like response sequences	-	√

*Note*. √: assumed or discussed, (√): briefly mentioned, -: not explicitly assumed or discussed

A central assumption of C-SMB 2.0 is that all learning, including the development of motor sequences and of executive control, is critically dependent on associations with the task, goal and sensory representations that were active during practice (Howard & Kahana, 2002; Logan & Cox, 2021). In the case of motor sequences, the representation of each sequence element at a particular processing level is activated by a context that consists of appropriate goal and task representations and the gradually fading activation caused by executing prior sequence elements (Logan & Cox, 2021).

**Fig. 2:**
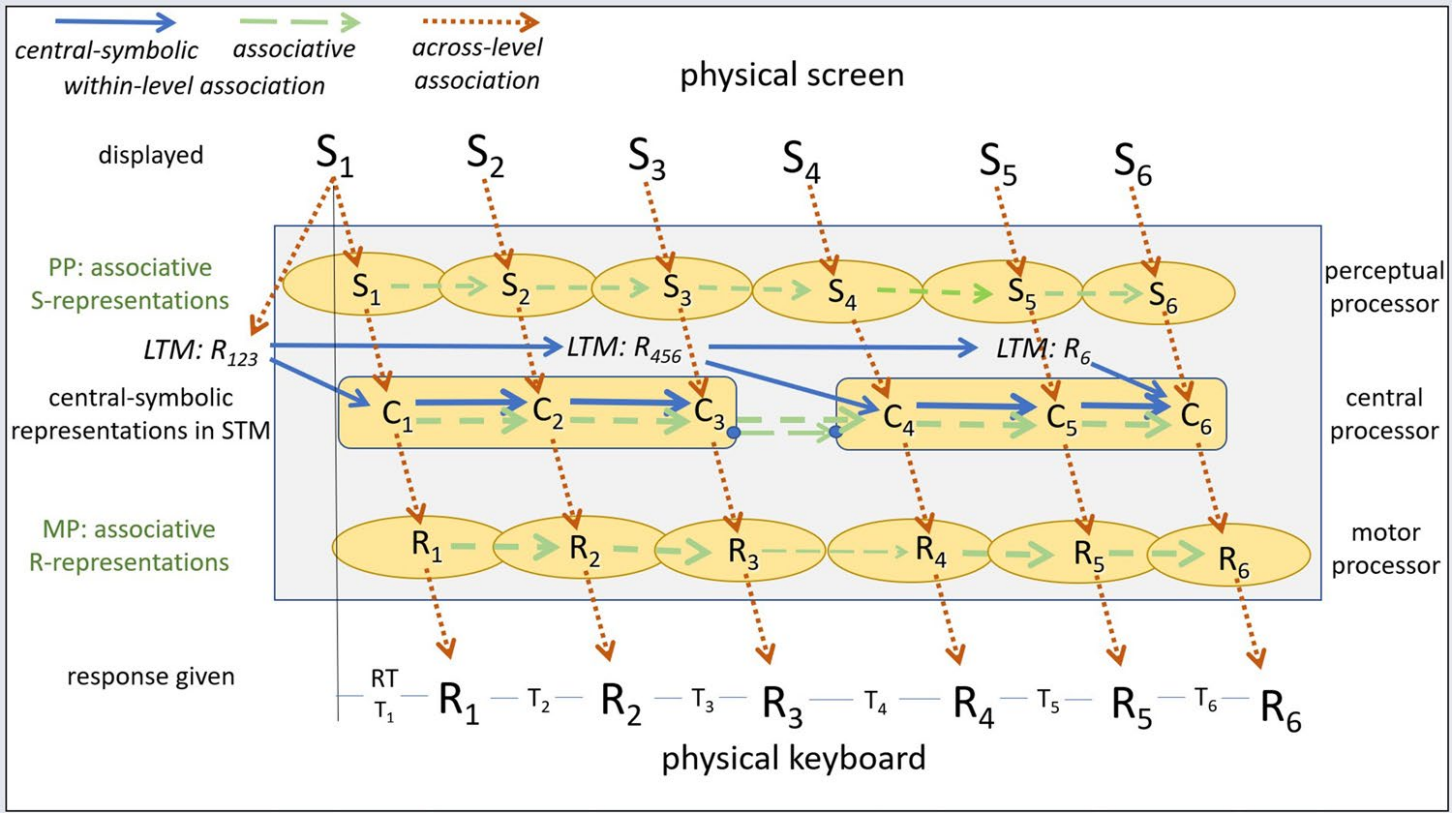
The four types of associations responsible for skilled execution of a 6-key DSP sequence according to C-SMB 2.0. (**1**) S–R associations allow for the rapid responding to key-specific stimuli by a dedicated part of the CP, CP_SR_ (red arrows). (**2**) Central-symbolic representations of segments consist of, here, three (spatial/verbal) abstract response representations (C_123_ and C_456_) that result from repeated activation in STM (blue arrows in rounded squares). (**3**) The transition between successive central-symbolic representation-based segments is indicated by the relatively slow concatenation response R_4_. This transition is initially controlled by the CP but with practice associations develop between successively used abstract central-symbolic representations and their responses (indicated by the green arrow with blue dots between the rounded squares). (**4**) Associations develop more slowly at the perceptual, central and motor levels between representations used successively at each of these levels (indicated by overlapping ovals at the perceptual and motor levels and the green arrows). Associations at the central and motor level gradually overrule the slow concatenation response and the application of central-symbolic representations. The relatively fast last response with moderate practice is attributed to the CP applying explicit, episodic knowledge that is prepared while the MP is executing the last segment (two blue arrows). Not indicated are the context effects caused by sensory feedback of preceding responses, and by the general task- and goal-specific representations that the CP prepares in advance of sequence execution. Note: S_1_-S_6_: key-specific stimuli and their perceptual representations; C_1_-C_6_: central item codes in STM; R_1_-R_6_: representations of Responses 1 through 6 at the motor level; T_1_-T_6_: intervals preceding responses R_1_-R_6_, respectively. STM: short-term memory, LTM: long-term memory. (Color figure online)

C-SMB 2.0 distinguishes four types of associations (Fig. 2). In addition to (1) S–R associations used for selecting individual responses that are responsible for the reaction skill, associations are responsible (2) for connecting (spatial and/or verbal) response representations in STM yielding a context-specific central-symbolic sequence representation, (3) for concatenating successively used central-symbolic representations, and (4) for STM-independent associative sequence learning at each processing level. The *cognitive loop*, already proposed in C-SMB (Fig. 2 in Verwey et al., 2015), is responsible for the successive selection by the MP of abstract response representations in STM that are abstract because they do not include concrete motor features. In C-SMB 2.0 the motor loop emanates from associations at the motor level that concurrently trigger a succession of effector-specific response representations. Consistent with the notion that practice shifts the processing load from central to motor processing, central-symbolic representations are assumed to develop more rapidly than associative learning at the motor level. Details of associative sequence learning have been worked out in great detail in competitive queuing models (Bullock, 2004; Burgess & Hitch, 1999; Kornysheva et al., 2019; Mantziara et al., 2021, see the section “Computational models of sequence control”).

The distinction between the CP and the MP is consistent with two hierarchical control levels of which the highest level controls the order of the central-symbolic representations. According to C-SMB 2.0, initially this concatenation is a function of the CP, but after hundreds of practice trials, this is achieved by associations between successive central-symbolic representations as a whole. The hierarchically lower level involves the MP controlling the order of individual responses within central-symbolic representations that have already been prepared in STM. The use of two hierarchical control levels when executing DSP sequences is consistent with studies on keying and speech that did not found evidence for more than two hierarchical control levels (Logan & Crump, 2011; Sternberg et al., 1990; Yokoi & Diedrichsen, 2019).

A few DSP studies suggest that the order in which the CP performs processes—a process indicated as executive control—is supervised in the same way as the individual responses in a motor sequence. That is, the order of both responses and processes can be prepared in advance and consistent use of a specific processing order automates with practice (Verwey & Abrahamse, 2012; Verwey et al., 2010, 2014). Automation of the order of the processes that are carried out by the CP is suggested by the slowing observed when in a practiced task a deviating processing order is required. The remainder of this section presents the various assumptions of C-SMB 2.0 that are suggested by the review of DSP studies in “Reviewing DSP task results.”

#### Lasting use of key-specific stimuli

A first new assumption of C-SMB 2.0 is that instead of the increasing independence with practice from key-specific stimuli suggested by C-SMB, the luminance change associated with the display of key-specific stimuli continues to attract attention and triggers the associated response (Verwey et al., 2020). This lasting reliance on key-specific stimuli is responsible for the limited ability of many participants to execute DSP sequences without key-specific stimuli after they practiced those sequences by responding to such stimuli. Stimulus-independent sequence execution can, however, be learned by practicing in situations in which the key-specific stimuli after the first do not capture attention. This is the case when these stimuli are not displayed at all during practice (e.g., Verwey et al., 2002, 2016), and also when key-specific stimuli are displayed only when the ensuing response is not given in time (Verwey, 2021). Participants might also learn to do without key-specific stimuli when stimuli after the first are isoluminant relative to the background so that they can be ignored (Riesenbeck, 2021), and when they practice executing sequences as fast as possible and ignore errors (Barnhoorn et al., 2019a, b).

In line with the distinction between sensorimotor and more strategic executive control, the triggering of responses by key-specific stimuli becomes independent of the CP with practice and operates via a shortcut, the *CP*_*SR*_* channel* (Verwey, 2021). This CP_SR_ channel operates via shared stimulus and response features and may also involve context-dependent associations between stimulus and response features that develop during practice (Berlyne, 1957; Fuster, 2004; Hommel, 2019; Hommel et al., 2001; Koechlin & Summerfield, 2007; Schumacher & Hazeltine, 2016) and representations of the intended movement end effects (Fuster, 2004; Hommel, 2019; Hommel et al., 2001; Schumacher & Hazeltine, 2016). The existence of CP_SR_ is consistent with the elimination of response selection as a processing bottleneck with extended practice (Strobach et al., 2013). In fact, the existence of CP_SR_ is consistent with a versatile CP in that participants can learn to distribute CP processing resources across different tasks (Verwey et al., 2014).

#### Central-symbolic representations

The development of central-symbolic representations is assumed to be caused by the repeated construction and co-activation in STM of the same series of up to 4 responses. That is, preparation consolidates temporary bindings in STM eventually forming the permanent, context-dependent associations that underlie representations in LTM (Herwig & Waszak, 2012; Verwey et al., 2015). Consolidation in STM explains also sequence learning due to mental practice and repeated preparation without actual sequence execution (Sobierajewicz et al., 2016), better learning in random than blocked practice in the Contextual Interference paradigm (Verwey et al., 2022b), and that the development of central-symbolic representations in STM is independent of sequence execution rate (Mantziara et al., 2021). These central-symbolic representations can further be associated with both a sequence-specific stimulus or the first of a series of key-specific stimuli, and also with a preceding central-symbolic representation.

Central-symbolic representations are akin to the earlier proposed multidimensional event files that are held responsible for the skilled selection of individual responses, and that would incorporate the conceptual and intentional properties of stimuli and responses that are contingent on goals and contextual factors (Hommel, 2019, 2021; Hommel et al., 2001; Schumacher & Hazeltine, 2016). However, while these multidimensional event files are basically temporary representations constructed in STM, central-symbolic representations are supposed to consist of consolidated representations stored in LTM that, given the proper context, can be selected as a whole (Verwey, 1999), just like other memory ‘chunks’ (Gobet et al., 2016; Miller, 1956). Development of central-symbolic representations does not increase the number of responses that fit the STM (Ariani & Diedrichsen, 2019) because, once activated in STM, the CP still has to prepare these abstract sequence representations into the constituting abstract response representations in STM.

The responses retrieved from a central-symbolic representation are represented in the spatial and verbal codes for which STM has been argued to have different components (Baddeley, 2000). Yet the use of verbal representations, like the letters of the keys pressed, may be quickly abandoned as it involves no information that can be immediately applied. Instead, central-symbolic representations have been found to include hand-specific (trunk- or head-based, and possibly also other) spatial representations that can be easily applied to select individual responses (De Kleine & Verwey, 2009; Groen et al., 2022).

#### Segmentation

Another assumption of C-SMB 2.0 is that with normal 6- and 7-key DSP sequences, when each response is indicated by a key-specific stimulus, execution by the MP of the prepared first three or four responses is initially followed by the CP selecting in the reaction mode the ensuing responses on the basis of either the key-specific stimuli or reading explicit sequence knowledge from LTM. Eventually, these later responses are prepared together in STM, too, thus stimulating the development of further central-symbolic representations. The limited capacity of STM is therefore solely responsible for the development of segmentation of longer sequences. In the case participants rely on explicit letter or number series that have been learned in advance, segmentation shows up more rapidly in sequence execution than when reacting to key-specific stimuli because these explicitly learned verbal representations have already been segmented in STM. After a few hundred practice trials segmentation usually benefits sequence execution time but with still more practice this benefit reduces because associative sequence learning becomes dominant. Yet the reduced development of associations across the relatively long intervals between successive central-symbolic representations implies that even associative sequence learning initially mimics STM-induced segmentation.

The precise way in which longer DSP sequences are segmented usually shows considerable individual differences. Segmentation appears influenced by individual STM capacities but also by task properties like the pauses in prestructured sequences, the occurrence of conspicuous events and regularities in element order like runs, trills, and reversals. Still, a few specific DSP sequences appeared to show similar segmentation across participants even though keys had been counterbalanced across sequence positions for different participants (see below).

#### Associative learning outside of STM

C-SMB 2.0 assumes that executing DSP sequences induces associative sequence learning at the perceptual, central, and motor levels of processing. This learning is based on the development of associations between successively used representations at the various processing levels that develop when the same series of responses is given over and over again (Cohen & Sekuler, 2010; Howard & Kahana, 2002; Logan, 2018, 2021; Logan & Cox, 2021; Perlman et al., 2010). These associations are independent from preparation in STM and prime the processes and/or representations needed to produce each next response (as we know from the SRT task, e.g., Abrahamse et al., 2010; Keele et al., 2003). Unlike central-symbolic representations, these associations develop faster at higher execution rates because successively used representations are more co-activated. In DSP sequences associative sequence learning allows activation to accumulate across the successive responses and this results in an increasing independence of key-specific stimuli towards the sequence end. In a context of activated task and goal representations the association of successive representations eventually leads to autonomous sequence execution once the first one or two responses have been executed under control of the CP.

With many hundreds or even thousands of practice trials segmentation reduces because associations develop between successive responses, and this gradually overshadows the slow concatenation of successive central-symbolic representations. Associative learning at the motor level is held responsible for the effector-specific component in skilled motor sequence execution. That component may be responsible also for optimizing muscle-onset times which takes into account the effectors’ inertia and resting positions, thus allowing for coarticulation in speech, typing and playing musical instruments (Park & Shea, 2003; Shea & Kovacs, 2013; Sosnik et al., 2004). While not properly investigated in DSP studies, studies with the SRT task suggest that associative sequence learning also develops at the perceptual and central processing levels (Abrahamse et al., 2010; Goschke & Bolte, 2012). Pilot studies with the DSP task suggested that relative stimulus positions may even be learned at the retinal level (Giusti, 2021; Rödig, 2009), most likely because key-specific stimuli are detected and processed more rapidly.

#### Context effects

An important further assumption that is often overlooked is that sequences may become dependent on relevant, and even irrelevant, aspects of the situation (Berlyne, 1957; Koechlin & Summerfield, 2007). This context dependency may however be limited because participants learn to focus on relevant stimulus features and suppress irrelevant stimuli. Only when there is insufficient time to suppress irrelevant stimuli, or when these stimuli continue to attract attention, irrelevant stimuli may become integrated in sequence representations. This shows up as slowed sequence execution in a context that previously occurred with a competing sequence (Ruitenberg et al., 2012a, b, 2015). Context stimuli that co-vary with the relevant stimuli, like sensory feedback of responses given, are likely to also become integrated in the sequence representations and assist in sequence control (as shown in the SRT task by way of response effect learning; Ziessler & Nattkemper, 2001).^8^ A few DSP studies further suggested that previously activated task and goal representations may eventually contribute to activating required processes, and therewith contribute to executive control (Verwey et al., 2010, 2014).

#### Preparing DSP sequences

C-SMB 2.0 assumes that response preparation can occur at two processing levels. This would involve, first, preparing abstract representations of up to 4 responses in STM, one by one or together as a central-symbolic representation, and, second, by preparing a fully specified representation of a single response at the motor level. In simple-RT conditions, preparing the first response at the motor level can account for the reducing effect with practice of sequence length (Sternberg et al., 1978). That is, participants learn to prepare, before display of the go stimulus, a sequence in STM and concurrently specify a single response at the motor level. When the go stimulus is identified, participants immediately execute the first, fully specified response, which therefore is no longer subject to STM scanning (Rosenbaum et al., 1983; Sternberg et al., 1978) or internal competition (Bullock, 2004; Burgess & Hitch, 1999; Kornysheva et al., 2019). In choice-RT conditions, the sequence length effect is usually not observed because the various alternative first responses are all stored in STM (e.g., Cisek, 2007), and the required first response is immediately executed upon identification of the imperative stimulus because later sequence elements have not yet been stored in STM. In these choice conditions the second and later responses of the selected sequence are activated in STM by the CP only while the MP is executing the first response.

The repeated observation that in go/no-go DSP tasks more than 4 responses can be prepared (e.g., De Kleine & Van der Lubbe, 2011) is also attributed to preparing the second through fifth responses in STM while the already prepared first response is immediately executed. With even more responses participants might even learn to store the responses in different STM components (Baddeley, 2000) and prepare the next segment during execution of the preceding segment. For instance, while the responses of the first segment of a to-be-typed phone number are prepared in terms of key locations later numbers are initially stored in STM in a verbal code.

#### Concurrent processing

The possibility of the MP to autonomously execute responses—retrieved from STM or activated via associations with previous response representations—allows the CP to concurrently perform other processes. This may involve selecting the next central-symbolic representation but also the processes needed for some secondary task. The CP can further use explicit sequence knowledge and/or key-specific stimuli to speed up the last segment and especially its last response in the race with the MP. Nevertheless, with many hundreds of practice trials, associative sequence learning gradually overshadows this contribution of the CP to the last responses.

The concatenation response is assumed to be relatively slow because preparing in STM the individual responses on the basis of a selected central-symbolic representation cannot be done during execution of the preceding segment because that still occupies STM. Slowness of concatenation responses, and of the preceding segment, therefore shows that central-symbolic representations can be selected as a whole while earlier responses are executed, but response preparation in STM is possible only after the last response of the preceding segment has been executed (Sternberg et al., 1978).

#### Explicit sequence knowledge

Given the multimodal representation of DSP sequences it is quite obvious that the knowledge stored is influenced by the task at hand. When awareness tests are performed episodic memories may be accessed and implicit knowledge may be used for making sequence order explicit. This reconstruction using implicit knowledge involves playing off the sequence mentally or physically on the tabletop. Instead, the execution of DSP sequences relies heavily on the rapidly available knowledge in central-symbolic and associative sequence representations as retrieval of episodic knowledge and reconstruction of implicit knowledge is too slow. The higher execution rates of more aware participants executing familiar sequences appear to occur primarily after, say, a hundred practice trials when execution rate still allows the application of explicit knowledge. The finding that more-aware participants are faster on novel and random sequences too indicates that these participants also have better processing skills and/or are more motivated, which is probably why they also developed more explicit knowledge.

Right from the start of practice, participants develop awareness of especially the first few and last responses of a keying sequence. This knowledge is quickly used for preparing the first few responses in STM and also for selecting the last response during execution of its predecessors. This strategy most likely also contributes to how the sequence is segmented. Explicit sequence learning basically occurs unintentionally, but participants can improve their explicit sequence knowledge during practice by intentionally developing and testing hypotheses on the order of the sequence elements. Spatial knowledge of the ensuing stimuli and/or responses that develops with moderate practice in STM is well-suited for executing DSP sequences but need not be explicit (cf. Keele et al., 2003). Instead, verbal sequence knowledge—consisting for example of the letters of the keys pressed or numbers indicating stimulus or key locations—is by definition explicit. However, as especially verbal sequence representations in STM do not contain concrete response features, verbal sequence representations may not develop or are rapidly forgotten (Verwey et al., 2022a).

#### General skills

When practicing DSP sequences, participants also develop more general skills that benefit other DSP sequences, too. These skills seem responsible for musicians and video gamers executing DSP sequences better in early practice. The development of a general reaction skill is indicated by practice of DSP sequences benefiting even random keying series and unfamiliar sequences. Improved selection skill also benefits the initiation of DSP sequences, and most likely the first response, when prior practice involved responding to sequence-specific stimuli. Another general skill concerns the capacity to execute fixed DSP sequences in general. This is observed as a greater benefit from practicing familiar sequences when executing novel than random sequences. This task-specific, sequence-unspecific skill may involve the capacity to have the CP process information concurrently with the MP. Finally, in sequences executed at submaximal rates rhythms are learned independently from the sequence and can therefore be regarded a more general skill, too.

### Four phases of skill development

**Fig. 3:**
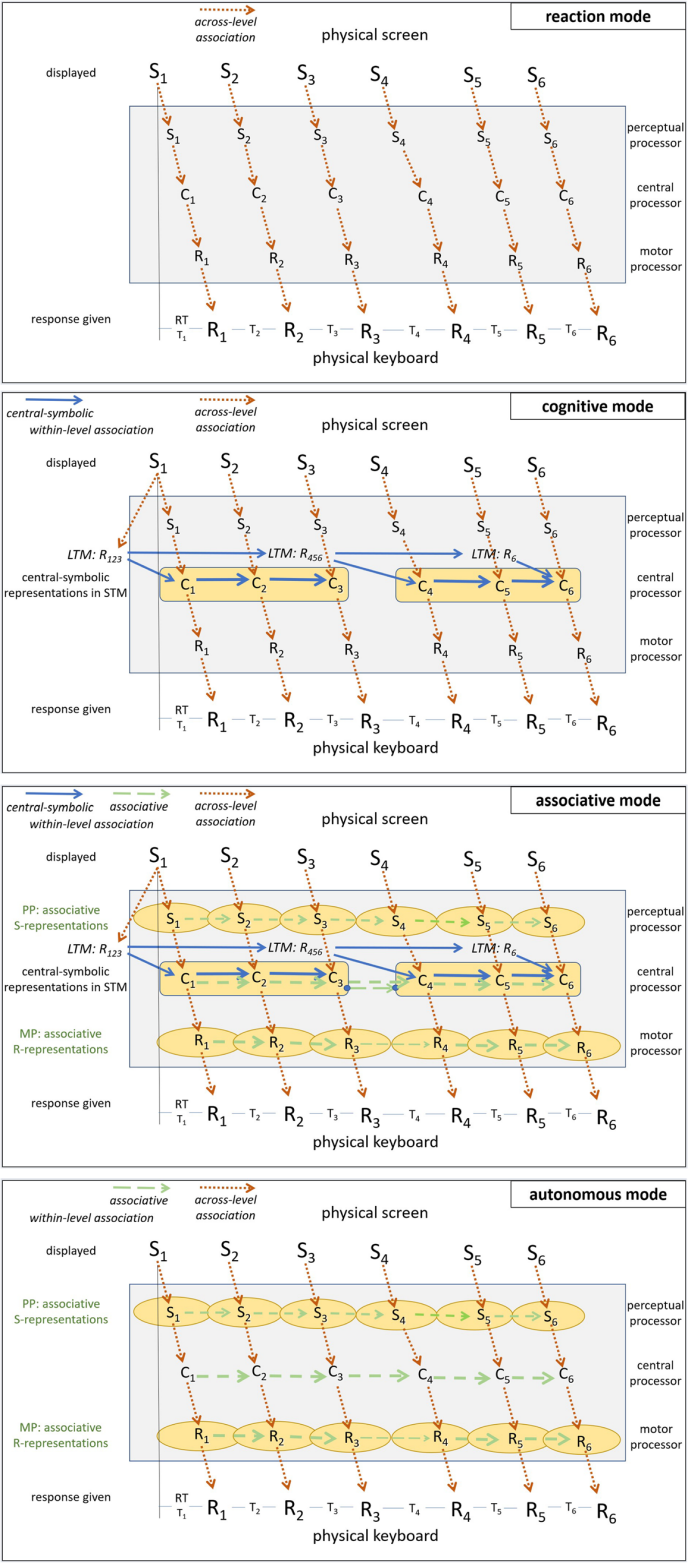
Involvement of the processors and associations in the reaction, the cognitive, the associative, and the autonomous execution modes (based on Fitts & Posner, 1967, and Fig. 2). (Color figure online)

A well-known classic model distinguished three modes of skill acquisition (Fitts & Posner, 1967). It postulates that during the *cognitive mode* participants require conscious attention to execute movements, and they are relatively slow and inaccurate. With practice, participants transition to the *associative mode*, which is characterized by improved accuracy and reduced reliance on conscious control. Finally, in the *autonomous mode*, movements become automatic, fast, and accurate, requiring minimal conscious effort. This classic model of skill development was a loose interpretation of everyday observations and data obtained when studying the acquisition of aimed movement skill. The present assumptions of C-SMB 2.0 on how processing changes in the course of practice allow a detailed account of the processes occurring in these three modes in the DSP task (Fig. 3), which are preceded by the *reaction mode*.

1. The reaction mode occurs during the first 10 to 20 practice trials of motor sequencing tasks. Participants operate by responding to key-specific stimuli. They then also learn the task goal and acquire explicit knowledge of the first few and last responses of each sequence.
2. During the next, say, 20 to 100 practice trials participants perform in the cognitive mode. In short sequences, they learn to develop and use their knowledge of the first two and eventually of more responses by preparing them in STM before initiating the sequence. In longer sequences, this prompts segmentation because the central-symbolic representations that develop in STM cannot include more than about 4 responses. These responses are facilitated by a context consisting of task and goal information and activations resulting from executing the preceding responses. While responses are executed by the MP the CP can prepare the oncoming segment, and later also focusing on the last response using explicit knowledge. Due to the lasting attentional capture of key-specific stimuli direct S–R associations develop and this also allows for concurrent response facilitation by the individual key-specific stimuli.
3. After a few hundreds of practice trials associations become increasingly important in the associative mode. In addition, the consistent execution of central-symbolic representations in a particular order allows the successive central-symbolic representations to become associated. Initially, sequences remain segmented because associations develop more slowly across the relatively long intersegment intervals. Executing the first few responses in the context of the active task and goal representations—previously set by the CP—then suffices to execute later sequence items by the MP without much further CP involvement. Concurrently, the repeated execution of the sequence induces associations between successively used representations at the motor, and probably also at the perceptual and central processing levels. This then primes the successive activation of these response representations and speeds up the execution of the ensuing responses. Such associative sequence learning is especially obvious at the motor level which shows by the development of the effector-specific sequence learning component and coarticulation. Onset of key-specific stimuli continues to trigger each next response too.
4. After many hundreds and even thousands of practice trials associative sequence control becomes dominant in the autonomous mode and execution becomes largely independent of central-cognitive processes. Indications for segmentation vanish because central-symbolic representations no longer important contribute much to sequence execution, and deliberate execution of the first response in the appropriate task context suffices for triggering the autonomous execution of the ensuing movements in the familiar sequence, leaving the CP free for other processing tasks. The contribution of key-specific stimuli may reduce due to the high execution rate at which the successive responses are executed and because of reduced visual attention to the key-specific stimuli.

### The validity of C-SMB 2.0

Given the flexibility of a general framework like C-SMB 2.0 to explain a large variety of results, validation of C-SMB 2.0 requires a broad research program that tests the basic phenomena and core assumptions (much like the TEC framework that was validated by replicating its six most important phenomena; Janczyk et al., 2023). The main phenomena that gave rise to C-SMB include the slow first response, the relatively slow concatenation response, and the slowing of a segment when followed by another segment. As discussed earlier, there is ample evidence for these phenomena. A core assumption is that processing is carried out by a few processors, each of which is responsible for specific processing stages and this allows preparation of oncoming responses and segments during execution of preceding responses and segments. The DSP task has provided ample evidence for concurrent preparation and evidence for concurrent activity of individual processing stages in the choice-RT paradigm has been provided by the PRP paradigm (e.g., Pashler, 1994; Sigman & Dehaene, 2005). The core assumption that learning is associative is based on the various indications that sequence learning occurs also without advance preparation and that repeated preparation yields central-symbolic representations.

Nevertheless, the assumptions in section “Introducing C-SMB 2.0” that are specific for C-SMB 2.0 sometimes still require empirical support. This research could, for example, include a line of research to further test the prediction that sequence performance reduces somewhat when one of the proposed sequence control mechanisms is no longer involved in the race of processors and the remaining mechanisms are solely responsible for sequence execution. That is, sequence execution should be slowed by removing or changing key-specific stimuli (as this would block the CP_SR_ contribution), by including a secondary task, like tone counting, and by reducing the time to prepare sequences (which should reduce the CP contribution), and by having participants use other fingers (which should block motor level sequence associations). This slowing might increase when in this way two sequence learning mechanisms are blocked. And depending on the mechanisms blocked segmentation might reduce or increase. Further tests of C-SMB 2.0 might explore the apparent capacity of participants to prepare more than 4 responses in go/no-go tasks. This is attributed to using different ways of coding the earlier and later responses (e.g., De Kleine & Van der Lubbe, 2011). And increasing the attention given to irrelevant stimuli during practice should result in strengthened susceptibility of sequencing skill to those context stimuli. Conversely, the reliance on key-specific stimuli should reduce when these do not capture visual attention during practice. Also, the assumption that motor sequences are represented by an integrated representation suggests that selection them involves activation of the anticipated distal effects of the entire motor sequence, just like with individual responses (Hommel et al., 2001; Prinz, 1997). Furthermore, when the individual responses making up a DSP sequence take more time to execute than just pressing a single key, indicators for segmentation are expected to reduce because longer lasting response execution conceals better the RT effects of concurrent processing. Yet, error rate may still show segmentation (Acuna et al., 2014). All in all, the merits of C-SMB 2.0 lie in its capacity to predict novel behavioral phenomena and eventually in its contribution to developing better, more detailed processing models for serial motor behavior.

### The neural foundation

As pointed out earlier (e.g., for emotion research; Barrett, 2006), progress in the study of the neural basis of behavior requires an inductive approach in which, first and foremost, a detailed description of relevant behaviors and the underlying processes is developed. C-SMB 2.0 is meant to provide this description for serial motor behavior. It allows neurophysiological research to go beyond listing active neural areas and networks when executing motor sequences at the various skill levels because C-SMB 2.0 provides proposals for their functionality.

Important supportive findings for the distinction between the perceptual, central and motor processors posited by C-SMB 2.0 comes from a network analysis including some 10,000 fMRI experiments (Bertolero et al., 2015) and a recent functional connectivity analysis (Wang et al., 2023). In line with C-SMB 2.0’s PPs and MPs, these studies demonstrate that primary sensory and motor regions have a locally clustered connectivity profile prompting informationally encapsulated modules. And consistent with C-SMB 2.0’s CP and the multidimensional central-symbolic representations (and also with the cognitive event and task files proposed by Hazeltine & Schumacher, 2016; Hommel et al., 2001; Memelink & Hommel, 2013), these two neuroimaging studies also showed evidence for task-specific modules that via connector hubs integrate information from across the brain via long-range connections.

In line with the distinction between CP and CP_SR_, action selection has been shown to occur more anteriorly in the prefrontal cortex as the selection is more instruction-based and relies less on an overlap between stimulus and response representations (Koechlin et al., 2003; Koechlin & Summerfield, 2007). Also, Bassett et al. (2015) found with a network analysis on fMRI data in a study with 10-key DSP sequences that had been practiced for up to 2,000 trials, that visual and motor neural systems were heavily integrated early in practice and that this integration reduced with practice. These findings are consistent with the reducing reliance on key-specific stimuli with practice through CP_SR_ as central-symbolic and associative sequence representations develop. Importantly, this study also showed that a greater individual reduction of this integration with practice correlated with improved sequence learning on later days of practice. So, better learners seem to have more quickly reduced their reliance on key-specific stimuli.

As to the often-heard complaint that the neural system does not distinguish between cognitive representations and processes, it can be speculated that representations constitute oscillatory activity across large parts of the brain (Helfrich & Knight, 2016) and that processing is caused by changing the attractor landscape of the brain’s allover neural network (cf. the global workspace postulated by Baars & Franklin, 2007; Franklin et al., 2005). Translating a representation into another one could then involve the transition of one into another oscillatory pattern due to an adjustment to the composition of the active neural network. These adjustments are probably controlled by the prefrontal cortex using active goal and context knowledge to determine what specific cortical areas should be connected and disconnected by the basal ganglia and hippocampus to this neural network (Freeman, 2007; Freeman & Rogers, 2003; Helfrich & Knight, 2016; Zylberberg et al., 2013). This idea about the execution of successive processes is consistent with the observation that oscillatory activity patterns pass through sequences of states, each of which lasting 100 to 300 ms (Roelfsema, 2005; Roelfsema et al., 1997; Varela et al., 2001). Such a mechanism might hold especially for central processes (like stimulus identification, response selection, and motor programming and motor loading; Verwey et al., 2015). The perceptual and motor processes are probably less flexible and occur within dedicated brain areas.

While these temporary neural network compositions may encompass many areas across the brain in the case of unfamiliar tasks, thereby allowing the involvement of broad multimodal representations, after extended practice, more efficient, task-specific networks develop that include less brain areas and yield leaner representations. This unburdens other brain areas that can then contribute to other processing networks and, hence, perform concurrent processes. This can account for the indications that the CP was eventually able to count target tones while stimuli were still triggering responses via the dedicated CP_SR_ response selection channel (Verwey et al., 2014).

To explore in detail the neural areas involved in learning and executing DSP sequences, we carried out an fMRI study in which unstructured and prestructured 4-key sequences were practiced for 500 trials each (Jouen et al., 2013; Verwey et al., 2019). The analyses were consistent with the hypothesis that a cortico-cerebellar network plays a specific role in the initial processing of temporal structure of the prestructured sequences while the basal ganglia play a role in acquiring the response order of the sequence (Jouen et al., 2013). Later analyses including unfamiliar and familiar unstructured sequences suggested activity in three functionally separate networks that seemed to coincide with three of C-SMB 2.0’s processing mechanisms (Verwey et al., 2019). (1) Reacting to key-specific stimuli in the reaction mode was associated with activity in a network comprising preSMA, bilateral dorsal premotor areas, and bilateral posterior parietal and precuneus. (2) The use of spatial central-symbolic representations in the cognitive mode seemed controlled by a left occipital-left temporal-bilateral posterior parietal network that triggers the premotor areas to select responses and that use a left inferior frontal-preSMA-cerebellar network for timing. (3) The associative sequence representation at the motor level, that C-SMB 2.0 assumes to become dominant with many hundreds of practice trials, seemed to rely on the sensorimotor cortico-subcortical loop that includes posterior striatum and SMA. As both primary and secondary motor areas were simultaneously active and these cortical areas are known to independently project to the spinal cord (e.g., Ghez & Krakauer, 2000), one could argue that development of C-SMB 2.0’s central-symbolic representations occurs in parietal networks that control individual movements via secondary motor areas (i.e., premotor cortex, supplementary motor area, and cingulate motor areas) projecting to spinal motor centers. And associative motor sequence learning seemed to occur in the primary somatosensory cortex (i.e., S1-M1), perhaps supported by learning at the spinal level (Adkins et al., 2006; Grau, 2014).^9^ The fMRI data were in line with the notion that executive control in motor tasks—attributed to C-SMB 2.0’s CP, too—involves lateral prefrontal cortex influencing the strength of communication between regions in the frontostriatal motor system (Miller & Cohen, 2001; Rae et al., 2015). The amount of practice in this study was most likely insufficient to reach the autonomous mode, but that execution mode may involve the development of cortico-cortical connections that make control by the basal ganglia superfluous (Ashby et al., 2007; Hélie et al., 2015).

The results of Verwey et al. (2019) are consistent with the race assumption of C-SMB 2.0 in that the areas assumed to be involved in reacting to key-stimuli and in applying central-symbolic representations were active together with the motor system that is assumed to be responsible for associative motor control. Neural activity also appeared higher in M1-S1 during the execution of unfamiliar than random sequences. This could be interpreted as an early indication for the development of effector-specific sequence representations and coarticulation in these areas. A right superior temporal activation was argued to indicate the selection of an integrated central-symbolic representation before its individual responses are activated in STM. Consistent with earlier findings (e.g., Chein & Schneider, 2005; Wymbs & Grafton, 2015), Verwey et al. (2019) further confirmed the brain-wide activity reduction with practice that is typically attributed to increased processing efficiency. All in all, this interpretation of fMRI data using C-SMB 2.0 is speculative but it indicates how cognitive models can guide the interpretation of neural activities across the brain.

## Related behavioral paradigms

This section briefly discusses various theories on motor sequence learning that inspired C-SMB 2.0. It starts off with research on the selection of individual responses, as this is at the basis of skilled motor sequence execution. Because with sequences of longer lasting movements, like serial pointing, pursuit motor, mirror tracing, and dynamic balancing tasks (e.g., Cook, 1933; Corkin, 1968; Knowlton & Schorn, 2022; Krakauer et al., 2019), the underlying cognitive processes are less likely to directly affect performance measures. This section focusses on sequencing studies in which the items consist of short-duration responses—namely, pressing a key and uttering a unit of speech, like a syllable or short word. Eventually, two computational models of sequence learning are discussed that provide credible mechanisms as to how sequences are learned at a single processing level. This section then shows that the basic processes and mechanisms in response selection and motor sequence learning of C-SMB 2.0 have been proposed in other studies before and that the innovation of C-SMB 2.0 is primarily in its processing architecture that allows concurrent sequence learning by developing central-symbolic representations in STM and associative sequence learning at various processing levels.

### Selecting responses

In C-SMB, the selection of individual responses has previously been attributed to S–R mapping rules in STM that with practice are replaced by S–R associations or separate instance representations in LTM (Duncan, 1978; Logan, 1988). In the C-SMB 2.0 framework, at the initial practice levels response selection is assumed to rely on the ideomotor mechanism described by the influential Theory of Event Coding (i.e., TEC; Hommel, 2019; Hommel et al., 2001; Prinz, 1997). TEC postulates that individual actions are initially coded at an abstract level by the distal features of the represented event which especially includes the effects of executing that action. This allows people to select goal-directed movements even though they have little conscious knowledge of their own motor system. This notion has some similarity with the notion that people select movements based on the intended final state of the effector used (Rosenbaum et al., 1990, 2012).

Another model of response selection also takes executive control processes into account. It seems to encompass TEC but emphasizes the role of task context and behavioral goals for response selection and seems to better allow for response selection at more advanced skill levels (Hazeltine & Schumacher, 2016; Schumacher & Hazeltine, 2016). It asserts that the response is selected that best satisfies the constraints set by the stimulus, the anticipated distal effects of the response, the environment, and the task goal (Fuster, 2004; Hommel et al., 2001; Logan, 2021; Pashler & Baylis, 1991; Schumacher & Hazeltine, 2016). Recent research confirms that, in line with C-SMB 2.0’s CP_SR_ channel, associations develop with practice between the most rapidly available feature of the stimulus and the response (Verwey, in press-b).

### Keying sequences

The most notable phenomenon observed in the production of short behavioral movement series concerns the increase in initiation time as these series become more complex (Henry & Rogers, 1960). This complexity effect occurs especially in simple-RT tasks and was later attributed specifically to the number of responses in the sequence (Rosenbaum et al., 1987; Sternberg et al., 1978). Depending on the sequence elements, this *sequence length effect* levels off with about 4 or 5 elements (Sternberg et al., 1978; van Mier & Hulstijn, 1993). The gradual reduction of this sequence length effect with practice in the simple-RT tasks and the usual absence of it in choice-RT tasks is accounted for by participants immediately executing the first response (Klapp, 2003; Portier et al., 1990).

Klapp (1995, 2003) focused with his dit-dah task on the preparation in simple- and choice-RT paradigms of 1 and 4 successive key presses, each of which lasting either 150 ms or 450 ms and of pseudowords with 1 to 3 syllables. RTs of the first response were explained by two preparation processes—the INT process, which prepares each individual response and takes longer as that response lasts longer, and the SEQ process, which prepares the order of abstract response representations and that takes longer with 4 successive responses than with 1 response. The results showed that simple-RT is slowed by the number of elements and not by the duration of the first response. This was taken to indicate that in simple-RT conditions preparation of the first response (by INT; Magnuson et al., 2004) as well as of the sequence (by SEQ) occur before identification of the go signal (Immink & Wright, 2001; Wright et al., 2004). The sequence length effect would be due to searching the first response among the prepared response representations (cf. Sternberg et al., 1978). In choice-RT conditions, the sequence is obviously prepared (SEQ) only after identification of the first stimulus. The first response is immediately executed after stimulus identification so that there is no sequence length effect while choice-RT is affected by duration of the first response (INT). The preparation of later responses (INT) occurs while the preceding responses are being executed. Just like C-SMB 2.0, this model emphasizes the distinction between preparing sequences of abstract response representations (SEQ) and preparing the detailed, motoric, preparation of individual responses (INT) that may concur with execution of earlier responses and that precludes a sequence length effect.

A sequence learning task that is quite similar to the DSP task is the SRT task. The DSP task has even been confused with the SRT task (Lin et al., 2016, 2018). The differences between the two tasks pertain especially to the possibility with DSP sequences to prepare responses in advance and the high awareness of the first and last responses. The SRT and DSP tasks usually differ also with respect to the length of the motor sequence (6 or 7 vs. 12 responses, respectively), the amount of practice (30–100 cycles vs. 500 trials), the number of alternative sequences (one vs. two ) and the response-stimulus interval (RSI 200 vs. 0 ms). These properties of the SRT task make participants continue to respond to key-specific stimuli while preparing and executing response segments usually does not occur (unless there are pauses or specific regularities; Jiménez et al., 2011; Koch & Hoffmann, 2000a; Stadler, 1993; Verwey, 1996). The continuous responding to individual stimuli makes the SRT task especially suited to study implicit sequence learning. This would involve the formation of associations between successively used stimuli and stimulus features (causing perceptual sequence learning), successively used responses and response features (causing response sequence learning), and successively used response-to-stimulus compounds (causing response effect learning; Abrahamse et al., 2010; Goschke & Bolte, 2012). According to Keele et al.’s (2003) dual system model implicit learning would occur in specific modules in dorsal parts of the brain. The development of explicit—that is, verbalizable—sequence knowledge in a number of the participants would occur in ventral brain parts, and is stimulated by detecting mismatches between implicitly expected and experienced behavior (Esser et al., 2022; Frensch & Rünger, 2003).

### Typing and speech studies

Sternberg’s pioneering work in the late 1970s through 1990 on sequence execution shed light on the information processes responsible for producing short, relatively unfamiliar typing and speech sequences in simple-RT tasks (Sternberg et al., 1978, 1980, 1990). According to Sternberg et al.’s (1978) still relevant subprogram retrieval model, initiating short motor sequences is preceded by a process that searches and retrieves the subprogram for each of the appropriate responses by a sequential self-terminating search process. This is then followed by unpacking the retrieved response after which commands are issued for its execution. This model, too, distinguished between executing individual responses and controlling sequence order. The unpacking process would involve the detailed specification of each response before it is executed. The sequence length effect in these simple-RT tasks was attributed to the motoric representation of the first response being subject to rapid decay so that the retrieval and unpacking processes can only follow the go signal once the first abstract response representation has been found in the buffer (cf. Canic & Franks, 1989; Klapp, 2003).

An early theory for skilled production of speech sequences was proposed by MacKay (1982). He pointed out that serial motor skill must be represented at several levels of abstraction because transfer of practice was partial when another muscle system was used, like when writing with the nondominant hand, and also when another language was used like when German–English bilinguals reproduce practiced sentences in their other language (MacKay & Bowman, 1969). MacKay (1982) further argued that the increased speed and flexibility of skilled performance seem contradictory. Increased speed suggests increasing reliance on a fixed motor program that may not even require feedback, whereas increased flexibility suggests that behavioral patterns involve small, discrete components that remain interruptible. To account for these and other observations in speech sequences, MacKay (1982) proposed that uttering sentences is controlled by a hierarchical system with nodes at eight levels, starting at the propositional concept level, and then via various phonological node levels down to the muscle-specific movement level. Each node in this hierarchical system represents a class of actions at varying levels of abstraction. This theory is important because it constitutes a nice example of how serial behavior may be simultaneously based in various levels of abstraction—from the conceptual down to the motor level (cf. Miller et al., 1960).

The obviously hierarchical nature of language suggests that skilled typewriting also involves hierarchically ordered processes (Sternberg et al., 1990). Indeed, Logan and Crump (2011) proposed a two-loop theory of skilled typewriting that postulates that typing involves two autonomous processing loops. The outer loop takes text to be typed and parses it into word series. These words are then used by the inner loop to determine the series of keystrokes needed.

## Computational models of sequence control

Various computational models have been developed over the years to understand how serial order may be controlled. However, in contrast to C-SMB 2.0, these models assume that sequence learning occurs at a single processing level. These models go beyond the single association between the sensory effect of the preceding element and the next sequence element in the classic reflex chaining models because items are triggered by entire contexts built from fading traces of several earlier items. This context-based retrieval mechanism requires higher-level control at the sequence beginning to initiate retrieval and at its end to determine what to do next, but sequence execution runs autonomously to completion once started.

Competitive queuing models postulate that preparatory activity preactivates several oncoming item representations concurrently in a neural network and that the activation pattern of each item is weighted according to its position in the sequence (Burgess & Hitch, 1999). It accounts, for example, for the sequence length effect by assuming greater competition as there are more sequence elements (Bullock, 2004; Burgess & Hitch, 1999; Kornysheva et al., 2019). For well-trained finger sequences, neurophysiological support for such control of sequence movements is that during sequence planning, neural activity indicated the order of the oncoming movements (Kornysheva et al., 2019; Pinet et al., 2019).

A related model of sequence learning is the context retrieval and updating (CRU) model proposed by Logan (2018) for typing the short letter series in the inner loop of the Logan and Crump (2011) model. It asserts that serial order is controlled by a contextual matching process in which executing keystrokes builds up a representation of the context that activates oncoming sequence elements. Logan (2021) proposed that the associative mechanism of the CRU model is generally used for controlling serial behavior and he reported evidence that this mechanism underlies serial behavior in perceptual and memory tasks too. A single mechanism controlling serial order in various tasks is consistent with C-SMB 2.0’s core assumption that sequence learning is associative and occurs at various processing levels.

## Conclusions and final comments

Many years ago, Allen Newell (1973) warned the cognitive psychologists of his days that accumulating hundreds of individual phenomena ‘ad nauseum’ would not lead to a theory of cognition. That would require combining the many individual findings in single cognitive frameworks (that he later indeed developed; Newell, 1982; Rosenbloom et al., 1993). Many years later a similar worry was expressed by Gazzaniga (2010) when he wrote that neuroscientists “cling to the idea that understanding the elementary parts of the nervous system will explain how the brain does its magic to produce the psychological states we all enjoy” (p. 291). Today, an increasing number of researchers assert that limited theorizing is an important cause of the alleged crises in psychology (Eronen & Bringmann, 2021; Janczyk et al., 2023; Muthukrishna & Henrich, 2019). Given these recurring calls for better theories, I hope that the C-SMB 2.0 framework contributes to the development of a better understanding of the cognitive processes that underlie skilled motor behavior—not only to account for the various behavioral phenomena associated with serial motor skills but also for a better understanding of how the neural system processes information and produces behavior.

The present review reiterates the idea of the earlier C-SMB framework that performance in the DSP task can be accounted for by processors at the perceptual, central, and motor processing levels. These processors are assumed to be responsible for the processing stages suggested by choice-RT tasks and discrete sequencing tasks (Sanders, 1990; Sternberg, 1969; Verwey et al., 2015) and, importantly, also for the many indications for concurrent processing when executing DSP sequences. C-SMB 2.0 now explicitly states that learning in the DSP task occurs by both the repeated preparation of responses in STM and by the repeated co-activation of representations at each of the processing levels during actual sequence execution. Sequence learning seems a fundamental capacity of the nervous system that occurs in all its systems, perhaps even within the retina. C-SMB 2.0 also suggests that explicit knowledge as assessed by awareness tests results in part from reconstruction processes that are too slow to contribute to the rapidly executed motor sequences in the DSP task. It makes explicit the important role of the context for both the sequential and executive control of sequencing tasks and suggests that attention during practice determines what information is eventually being incorporated in the multidimensional representations that underlie sequencing skills. These notions can account for the flexibility and efficiency of motor skills (MacKay, 1982), for the resilience of these skills to secondary tasks and to deteriorated brain functioning caused by trauma and aging (e.g., Helmuth et al., 2000), and for the often-observed individual differences because the neural systems of people differ (Bo & Seidler, 2009; Verwey et al., 2022b).

An obvious limitation of C-SMB 2.0 is that it is mute about the role of feedback information. It is clear that sensory feedback is essential for skilled motor control, both at the level of individual responses as well as at the level of integrated motor skills, but this has not been explored with the DSP task. Another obvious limitation of C-SMB 2.0 is that it is based on results obtained with sequences of key presses. As argued earlier, there was a good reason for this, but future research will have to determine the merit of C-SMB 2.0 for other motor sequencing tasks, too.

Cognitive models are a reflection of the way the neural system behaves in specific situations. Future research should now look in more detail into how the cognitive processes and mechanisms assumed by C-SMB 2.0 are implemented in the nervous system. The proposed cognitive architecture seems to lend itself well to computational and neural modeling. The presented insights may eventually contribute to the building of more intelligent systems (e.g., Eliasmith, 2013; Kogut et al., 2014; Merolla et al., 2014). Following Richard Feynman’s lead that “what I cannot create, I do not understand” (Way, 2017), the ultimate proof that we understand how the nervous system produces goal-directed behavior should come from our ability to build computational models that use the processing architectures of our cognitive models and that show the behavioral phenomena observed in our studies.

## Glossary

Terms associated specifically with C-SMB 2.0 (and C-SMB) and the DSP task are in *italics*.

- *1x6 sequences*: 6-key sequences without any imposed pauses.
- *2x3 sequences*: 6-key sequences involving repetition of the same 3-key segment. This stimulates concatenation at R_4_.
- *2-2-2 sequences*: 6-key sequence practiced with pauses following R_2_ and R_4_ (Verwey et al., 2009).
- *3-3 sequences*: 6-key sequence practiced with a pause following R_3_ (Verwey et al., 2009).
- allocentric representation: a representation of a location in space relative to a reference point external to the body, like another object, the room, or geographic directions, such as North and South.
- *associative execution mode*: the faster execution of response sequences in which participants need not be aware of a fixed order and do not prepare segments or sequences (Verwey & Abrahamse, 2012; Verwey & Wright, 2014).
- *associative sequence representation*: A sequence representation that develops as a consequence of extensive practice by associating representations of successive movements. It differs from C-SMB’s motor chunks in that it is not limited to about 4 responses, and it can occur at perceptual, central, and motor levels of processing.
- *autonomous execution mode*: sequence execution based heavily on associations between representations at any processing level, as well as between effects of executing individual responses and successively used representations.
- awareness: performance of participants when writing down the letters of the keys pressed, or clicking with the mouse the locations or the letters of the keys they pressed when executing the practiced DSP sequence. E-Prime 2.0 source codes of the latter test are available on the Open Science Framework (https://osf.io/hmpnc/?view_only=c4e58cb5646e481d9775ebe86b0bc5ea).
- *C-SMB*: Cognitive framework for Sequential Motor Behavior, proposed by Verwey et al. (2015).
- *C-SMB 2.0*: improved version of C-SMB proposed in this paper. Features of C-SMB 2.0 are depicted in Fig. 2, and differences with C-SMB in Table 1.
- *Central processor, or CP*: Integrated processing system responsible for activating (spatial and verbal) central-symbolic representations in STM. It also is assumed to have an executive function as it prepares the task, goal, and context representations needed to activate the required processes and it is held responsible for the ‘central’ processing stages (Sanders, 1990; Verwey et al., 2015).
- *central-symbolic sequence representation*: an abstract, multidimensional representation that according to C-SMB and C-SMB 2.0 consists of up to about 4 element representations. It can be retrieved as a whole from LTM and activated by the CP as an abstract, spatial or verbal representation of the individual responses in STM. It develops as a function of repeated preparation in tens of practice trials.
- choice-RT task or condition: a task or condition in which one, and in DSP tasks several, responses are executed in response to one of a few alternative stimuli.
- chunk: a term used to indicate both a segment indicated by slow concatenation responses at the start, and the representation of it. This term was central in C-SMB and is no longer used in C-SMB 2.0.
- chunking mode: sequence execution based on employing motor chunks (in DPM and C-SMB)
- *cognitive mode*: sequence execution based on central-symbolic representations.
- *concatenation response*: the slow response that indicates the initiation of the next segment in sequences with over 4 or 5 responses.
- *DPM or dual processor model*: Model proposed by Verwey (2001) assuming a cognitive (now called ‘central’) processor and a motor processor. Worked out more in detail in Abrahamse et al. (2013).
- *DSP or discrete sequence production task*: a task typically involving the execution of one of two alternative keying sequences given in response to key-specific stimuli. DSP sequences typically include 3, 6, or 7 key presses. This task is central in the present review. E-Prime 2.0 source codes are available on the Open Science Framework (https://osf.io/hmpnc/?view_only=c4e58cb5646e481d9775ebe86b0bc5ea).
- egocentric representation: a representation of a location in space relative to some part of the body such as the eye, head, trunk, or wrist.
- *execution phase*: term used in some studies to indicate the various execution responses, as opposed to initiation and concatenation responses.
- *familiar sequence*: a DSP sequence that has been practiced and execution involves the use of central-symbolic and/or associative representations.
- flexion-extension or FE task: A task similar to the DSP task but participants move a lever with their forearm in response to displayed goal positions.
- interkey interval, or IKI: the time between successive key presses. With a 0-RSI IKI equals RT.
- *key-specific stimulus*: A stimulus, usually at a location spatially compatible with that of the required response. In DSP sequences, it is displayed following execution of the preceding key press to indicate the next key press.
- *location*: term used to indicate where in space a response is to be given (unlike ‘position’).
- LTM: Long-term memory, a central concept in cognitive research indicating long-term storage of knowledge. Activated knowledge in LTM is assumed to make up the STM content.
- M1: primary motor cortex
- motor buffer: according to C-SMB a short-term storage of up to about 4 movements that can be considered a component of STM. C-SMB 2.0 assumes that short-term storage of these responses occurs solely in (one of the components of) STM and that at the motor level only 1 movement can be prepared in detail.
- motor chunk: according to C-SMB a sequence representation in the motor buffer that is effector-specific, develops only after hundreds of practice trials and is assumed to include up to about 4 responses. Earlier, the term was used to designate all segment representations, irrespective of whether they occurred at the central-cognitive (verbal, spatial) or motor level. In C-SMB 2.0 this construct is not used.
- *motor processor, or MP*: processing system responsible for retrieving individual responses from STM and executing them. The MP allows detailed preparation of a single movement. With extensive practice, associations develop between response representations at this motor level which yields the associative sequence representation that is marked by the effector-specific component of sequence execution and coarticulation.
- *position*: term to indicate when in a DSP sequence a particular element occurs, sometimes denoted by a subscript (S_n_ or R_n_) (unlike ‘location’).
- *prestructured sequence*: a 6- or 7-key sequence practiced with a pause after R_3_, or after R_2_ and R_4_, in order to impose segmentation during practice.
- *R*_*x*_: response at sequence Position X.
- *random sequence*: discrete sequence in which each next stimulus is determined randomly except that immediate repetitions are prevented. Performance is assumed to reflect reaction skill.
- rate effect: the increase in the mean execution time of responses in longer sequences.
- *reaction mode*: executing a sequence by reacting to successive key-specific stimuli in the absence of sequence knowledge, used in unfamiliar and random sequences.
- reaction skill: the effect of practice on reacting to a stimulus with a single response.
- response: in choice- and simple-RT studies a ‘response’ usually involves the execution of a key pressing movement in reaction to a stimulus. In DSP sequences, ‘response’ may also refer to an individual movement that with practice no longer constitutes a reaction to a stimulus.
- RSI or response stimulus interval: Time between onset of a response onset and of the onset of the ensuing stimulus. In a DSP task RSI is typically 0 but in prestructured sequences one or two RSIs may be a few hundred ms to impose the same segmentation across participants.
- RT or response time: The time between onset of a stimulus and the associated response. As in familiar motor sequences a response need not always result from the preceding stimulus, in DSP studies the term ‘response time’ is preferred over ‘reaction time’.
- S1: primary somatosensory cortex (not be confused with the first stimulus, S_1_).
- *segment*: the behavioral manifestation of a central-symbolic representation. Referred to by some authors and in the first version of C-SMB as ‘chunk’ or ‘motor chunk’. The occurrence of segments in a DSP sequence is indicated by a relatively slow first response amidst fast responses, but segments are often concealed by individual differences. Slowing at the start of a segment is attributed to concatenating central-symbolic representations of up to 4 responses which results from preparing responses into STM. Segments may develop spontaneously due to STM limitations, but their development can be imposed also by practicing with a relatively long RSI and by regularities in the sequence.
- sequence length effect: the longer time taken to initiate sequences with up to 5 responses. Traditionally indicated by the more general ‘complexity effect’.
- serial reaction time (SRT) task: a sequence learning paradigm in which typically a fixed series of 12 stimuli is repeatedly cycled through and responses consist of pressing four keys with four fingers. In this task, there is only one sequence which is repeated about 30 to 100 times. Participants usually continue reacting to each stimulus and do not prepare segments. Improvement is attributed to implicit, associative learning though participants may also develop explicit sequence knowledge.
- simple-RT task or condition: a task in which one, or in DSP tasks several, responses are executed in reaction to a go-stimulus. The response is known in advance.
- *single-stimulus condition*: a condition in which participants execute the practiced DSP sequences in response to a single stimulus as key-specific stimuli after the first are no longer displayed.
- SMA: supplementary motor area.
- STM: short-term memory, a central concept in cognitive research which is generally assumed to include a spatial component (the visuospatial sketchpad), a verbal component (the phonological loop) and eventually also an episodic buffer that is controlled by a central executive (Baddeley, 2000). Its content is often assumed to consist of the knowledge in LTM that is active. In DSP tasks it assumed to contain up to about 4 abstract response representations.
- *S*_*x*_: stimulus at sequence Position X.
- *transition phase*: term used in some DSP studies to indicate the concatenation response.
- *T*_*x*_: response time in a DSP sequence at Position X.
- *unfamiliar sequence*: a DSP sequence that has not yet been practiced and that relies on reacting to each key-specific stimulus.
- *unstructured sequence*: a 6- or 7-key sequence practiced with no pauses between a response and the ensuing stimulus (i.e., only 0 RSIs) allowing segmentation to develop spontaneously. The term is used to emphasize the difference with a prestructured sequence.

## Footnotes

1 Typical E-Prime 2.0 source codes and footage is available on the Open Science Framework (https://osf.io/hmpnc/?view_only=c4e58cb5646e481d9775ebe86b0bc5ea).

2 I therefore prefer the neutral term ‘response time’ for RT instead of ‘reaction time’ (Verwey, 1996). The term ‘inter-key intervals’ (IKI) has also been used (e.g., Verwey, 2001; Verwey & Wright, 2004); When response–stimulus interval (RSI) is 0, IKI equals RT.

3 Some studies confusingly speak about Interleaved Practice (IP) to indicate Random Practice (RP), and Repetitive Practice (RP) when referring to Blocked Practice (BP).

4 I use the term STM, instead of working memory, to explicitly distinguish between the temporary memory function of STM without referring to the ‘central executive’ assumed by the working memory concept (Baddeley & Hitch, 1974). Still, I presume that STM includes verbal, spatial, and episodic components based on the temporary activation of representations in visuospatial, motor, and semantic long-term memory (Baddeley et al., 2011). STM is assumed to consist of all activated elements of long-term memory (Cowan, 2008; Schneider & Shiffrin, 1977).

5 The gradual execution rate increase across successive responses cannot be explained by the fast last response that has been observed only during the first, say, hundred practice trials and that is attributed to explicit sequence knowledge (see the Concurrent Programming section) because the gradual execution rate increase developed only with extended practice and affected more responses than just the fast last response.

6 Re-analyses of the DSP study reported by Verwey (2010) showed a 37-ms sequence length effect in a choice-RT condition, *F*(1, 55) = 6.14, *p* = .03, η_p_^2^ = .10.

7 These findings actually suggest a tool to determine the order of processing stages (Verwey et al., 2015) in that slowing of responses in a DSP sequence can show when particular processes occur. This cannot be determined with the additive factors method (Sanders, 1990; Sternberg, 1969).

8 An interesting observation is that upon asking the AI chatbot ChatGPT 4 it indicated to use “a type of neural network designed to generate human-like text by predicting the next word in a sequence of text based on the context of the previous words.” That such powerful chatbots are also based on context-dependent information demonstrates the merit of context-dependent associations in C-SMB 2.0 for generating serial behavior.

9 The direct cortical-spinal projections from primary and secondary motor cortical areas suggest that in C-SMB 2.0 (Fig. 2) the CP does not trigger response representations in MP but bypasses the MP and directly triggers individual responses represented in the spinal cord.
